# The Rising Aerogel Fibers: Status, Challenges, and Opportunities

**DOI:** 10.1002/advs.202205762

**Published:** 2023-01-19

**Authors:** Zhizhi Sheng, Zengwei Liu, Yinglai Hou, Haotian Jiang, Yuzhen Li, Guangyong Li, Xuetong Zhang

**Affiliations:** ^1^ Suzhou Institute of Nano‐Tech and Nano Bionics Chinese Academy of Sciences Suzhou 215123 China; ^2^ Division of Surgery & Interventional Science University College London London NW3 2PF UK

**Keywords:** aerogel fibers, nano‐confining functionalization, nanoscale building blocks, spinning kinetics, spinning thermodynamics

## Abstract

Aerogel fibers garner tremendous scientific interest due to their unique properties such as ultrahigh porosity, large specific surface area, and ultralow thermal conductivity, enabling diverse potential applications in textile, environment, energy conversion and storage, and high‐tech areas. Here, the fabrication methodologies to construct the aerogel fibers starting from nanoscale building blocks are overviewed, and the spinning thermodynamics and spinning kinetics associated with each technology are revealed. The huge pool of material choices that can be assembled into aerogel fibers is discussed. Furthermore, the fascinating properties of aerogel fibers, including mechanical, thermal, sorptive, optical, and fire‐retardant properties are elaborated on. Next, the nano‐confining functionalization strategy for aerogel fibers is particularly highlighted, touching upon the driving force for liquid encapsulation, solid–liquid interface adhesion, and interfacial stability. In addition, emerging applications in thermal management, smart wearable fabrics, water harvest, shielding, heat transfer devices, artificial muscles, and information storage, are discussed. Last, the existing challenges in the development of aerogel fibers are pointed out and light is shed on the opportunities in this burgeoning field.

## Graphical abstract

Aerogel fibers emerge as rising stars in diverse fields of thermal management, smart wearable fabrics, water harvest, shielding, heat‐transfer devices, artificial muscles, and information storage. Starting from nanoscale building blocks, spinning thermodynamics and spinning kinetics associated with a variety of technologies are revealed. Aerogel fibers will earn an irreplaceable place with the advances in materials and fabrication methodologies.

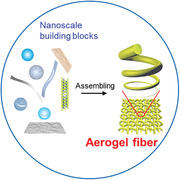

## 1 Introduction

Aerogels are pronounced porous materials with an ultralow density, ultrahigh porosity, large specific surface area, and extremely low thermal conductivity. The porous network is built of mesopores (2–50 nm) as well as macropores (>50 nm).^[^ ^1^ ^]^ Due to the unique porous structure and properties, aerogels have earned an unprecedented place in the fields of thermal insulation,^[^ ^2^, ^3^, ^4^ ^]^ environmental treatment,^[^ ^2^, ^5^ ^]^ energy storage and conversion,^[^ ^6^ ^]^ catalysis,^[^ ^7^ ^]^ biomedicine,^[^ ^8^ ^]^ electromagnetic absorption/shielding,^[^ ^9^ ^]^ and so on. They have found applications as the insulating material aboard the Mars Pathfinder rover, and in collecting the comet dust aboard the Stardust Spacecraft. A layer of ≈2–3 cm‐thick silica aerogel has been proposed to induce a local and potentially habitable subsurface environment on Mars via the solid‐state greenhouse effect.^[^ ^10^ ^]^ Aerogels are obtained by replacing the liquid in gels with a gas, eliminating the collapse of the gel solid network. This process involves three steps: solution–sol, sol–gel, and gel–aerogel transitions. Basically, there are two strategies for the sol–gel transition. On the one hand, precursor molecules undergo a hydrolysis reaction followed by a condensation reaction.^[^ ^11^ ^]^ On the other hand, the sol–gel process is achieved via the direct assembly of nanoscale building blocks into a macroscopic structure.^[^ ^12^ ^]^ This revolutionary strategy is prone to making full use of the rich toolbox of nanoscale materials, including 0‐dimensional (0D) nanoparticles,^[^ ^12^ ^]^ 1‐dimensional (1D) nanowires/nanotubes (polymer chains,^[^ ^13^ ^]^ carbon nanotubes,^[^ ^14^ ^]^ zircon nanofibers,^[^ ^4^ ^]^ etc.), and 2‐dimensional (2D) nanoflakes (graphene,^[^ ^15^ ^]^ MXene,^[^ ^16^ ^]^ Boron Nitride,^[^ ^17^ ^]^ etc.). Therefore, aerogels are considered the bridge between the nanoscale and the macroscale worlds, and their multitude of applications is almost unbounded.

**Figure 1:**
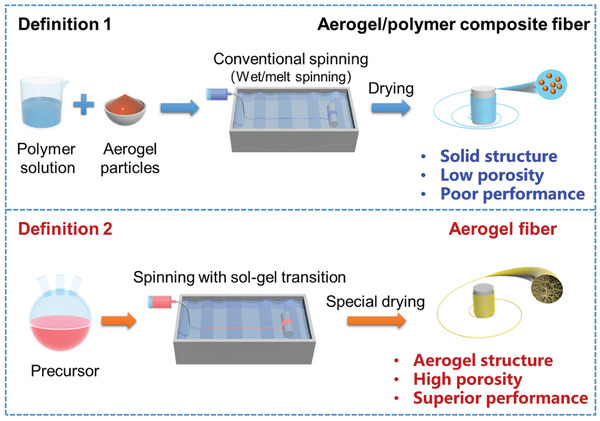
Schematics of aerogel fiber with the precise definition (definition 2), which is distinguished from the so‐called aerogel/polymer composite fiber (definition 1). The images of “Conventional spinning,” “Spinning with sol‐gel transition,” and “Aerogel fiber” are: Reproduced with permission.^[^ ^13^ ^]^ Copyright 2019, American Chemical Society. Other components in the figure are self‐produced by the authors.

Despite the vast development of aerogels in various fields, conventional sol–gel transition tends to be a static and slow process, which is difficult to be combined with the fast, dynamic, and continuous fiber spinning process. Hence, the current macroscopic aerogels are mostly monoliths with brittleness and poor shape fidelity, while the integration of diverse morphology, improved machinability, and multiple functionalities are greatly needed.^[^ ^9^, ^15^ ^]^ In this regard, aerogel fibers with superb flexibility, weavability, and easy integration capability, come to the forefront.^[^ ^18^ ^]^ Basically, two definitions related to aerogel fibers exist. Definition 1 of the aerogel fiber refers to the process that involves wet/melt spinning of polymer solution mixed with a slight amount of silica aerogel particles and followed by drying.^[^ ^19^, ^20^ ^]^ The resulting construct is the so‐called aerogel/polymer composite fiber, which is a solid structure with low porosity and poor performance. However, whether the porous structure in the aerogel particles is retained in the final fiber remains arguable as the pores can be occupied by the polymer solution during the wet spinning process or may collapse because of the high pressure during the extrusion process. Therefore, it is necessary to verify this point by the characterization of BET surface area or the true density of the fiber. On the other hand, definition 2 of the aerogel fiber refers to the procedure utilizing the nanoscale building blocks of particular materials, spinning the precursor with sol–gel transition, and special drying in sequence.^[^ ^9^, ^13^, ^15^, ^16^, ^21^, ^22^ ^]^ Through this strategy, the aerogel structure with high porosity and superior performance can be achieved (**Figure** **1**). Basically, for the current aerogel fibers, the range of pore size is ≈2–50 nm, the porosity is ≈95–99%, and the surface area is ≈200–1000 m^2^ g^−1^ depending on the huge variety of aerogel fiber materials and fabrication procedures.^[^ ^9^, ^13^, ^23^, ^24^ ^]^ Here, definition 1 is outside the scope of this review, and we mainly focus on the aerogel fiber derived from definition 2. In this regard, aerogel fibers are new types of high‐performance porous fiber materials, possessing a 3‐dimensional (3D) continuous porous network as well as fiber morphology. Owing to the inherent porous nature, the aerogel fibers exhibit unique heat transfer and mass transfer behavior, and a series of unprecedented properties.

So far, aerogel fibers have witnessed tremendous advances, including the assembling strategy of nanoscale building blocks and the abundant selections of materials. Depending on how fast the building blocks can be structured, that is, undergoing a static or dynamic sol–gel transition, spinning methodologies with variant spinning velocities have been developed.^[^ ^13^, ^21^, ^25^ ^]^ Different from bulky aerogels, aerogel fibers can be architected with tailored mechanical properties that can be bent, knotted, twisted, woven, and even designed as artificial muscles.^[^ ^14^, ^26^ ^]^ Being properly collected into aggregates or neatly woven into textiles, aerogel fibers are popular materials for super thermal insulation as wearable clothing.^[^ ^23^ ^]^ On the basis of the thermal insulation, multi functionalities have been sculptured with aerogel fibers,^[^ ^27^ ^]^ such as thermal/electrical/optical response,^[^ ^9^, ^15^, ^16^ ^]^ hydrophobicity,^[^ ^13^, ^21^ ^]^ sensing capability,^[^ ^28^ ^]^ fire‐resistance,^[^ ^21^, ^29^ ^]^ and washability,^[^ ^26^ ^]^ all of which have earned them an irreplaceable role in personal thermal management. In addition, the transparency of aerogel fibers can be maneuverable by controlling the aggregation degree of primary particles, as dominated by Rayleigh or Mie scattering.^[^ ^22^ ^]^ This is no wonder a giant step for the transparent wearable textile or even plenty of colorful clothing may come true depending on the assembly strategy.^[^ ^30^, ^31^ ^]^ Furthermore, aerogel fibers have been widely applied as adsorbents because of high porosity and high specific surface areas, such as moisture sorption or pollutant sorption.^[^ ^9^, ^32^ ^]^ Due to the significant capillarity and abundant micro/nano‐confined space, aerogel fibers can introduce a myriad of functional components into the pore structure, especially for functional liquids. To date, varieties of aerogel fiber confined solid‐liquid composites have been developed, endowing the liquid materials with good maneuverability, self‐supporting behavior, and programmable integration properties.^[^ ^13^, ^15^, ^26^, ^29^ ^]^ It is envisioned that aerogel fibers could be the future materials for personal thermal management, as well as personalized healthcare if incorporated with sensing and therapeutic capabilities.^[^ ^33^, ^34^ ^]^ Aerogel fibers can be applied for both military and civil uses, and the potential applications are almost unlimited.

**Figure 2:**
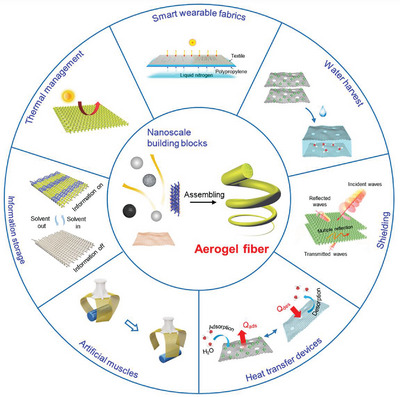
Overview of the aerogel fiber that is assembled from nanoscale building blocks, and the diverse fields of applications in thermal management, smart wearable fabrics, water harvest, shielding, heat transfer devices, artificial muscles, and information storage. The image of thermal management is: Reproduced with permission.^[^ ^13^ ^]^ Copyright 2019, American Chemical Society. The image of smart wearable fabrics: Reproduced with permission.^[^ ^15^ ^]^ Copyright 2018, Wiley‐VCH. Images of water harvest, shielding, and heat transfer devices: Reproduced with permission.^[^ ^9^ ^]^ Copyright 2022, Springer Nature. The image of artificial muscles: Reproduced with permission.^[^ ^26^ ^]^ Copyright 2021, American Chemical Society. The image of information storage: Reproduced with permission.^[^ ^35^ ^]^ Copyright 2022, American Chemical Society. The black porous sphere in nanoscale building blocks: Reproduced with permission.^[^ ^36^ ^]^ Copyright 2017, Elsevier B.V. The blue MXene in the nanoscale building blocks: Reproduced with permission.^[^ ^16^ ^]^ Copyright 2021, Wiley‐VCH. Other components in the nanoscale building blocks and aerogel fiber in the central area of the figure are self‐produced by the authors.

In this review, we examine the development of aerogel fibers rising in multiple fields. We summarize advances in fabrication techniques that produce the desired aerogel fibers, including the timeline, spinning dynamics, spinning kinetics, typical spinning methodologies, and dring techniques. We focus on the materials with tailored architectures that start the assembly from nanoscale building blocks. We describe the fascinating properties of aerogel fibers and highlight the nano‐confining functional strategy. We then explore the emerging applications they have enabled, specifically, thermal management, smart wearable fabrics, water harvest, shielding, heat transfer devices, artificial muscles, and information storage (**Figure** **2**). We end by discussing the existing challenges for both the fundamental science and industrial application, as well as offering a vision for what opportunities to capture in developing advanced aerogel fibers.

## 2 Fabrication Pathways Toward Aerogel Fibers

In this section, we overview the fabrication technologies of aerogel fibers. The timeline of the development of aerogel fibers regarding the material advances and fabrication pathways will be first touched upon. During the spinning of aerogel fibers, the spinning thermodynamics and spinning kinetics are two important factors determining the final fiber structure. Depending on the spinning speed, different strategies can be utilized, mainly falling into two categories: static sol–gel transition and dynamic sol–gel transition. Particularly, spinning methodologies including confined spinning, freeze spinning, wet spinning, reaction spinning, liquid crystalline spinning, and microfluidic spinning are discussed in detail.

### 2.1 Timeline of Aerogel Fibers

**Figure 3:**
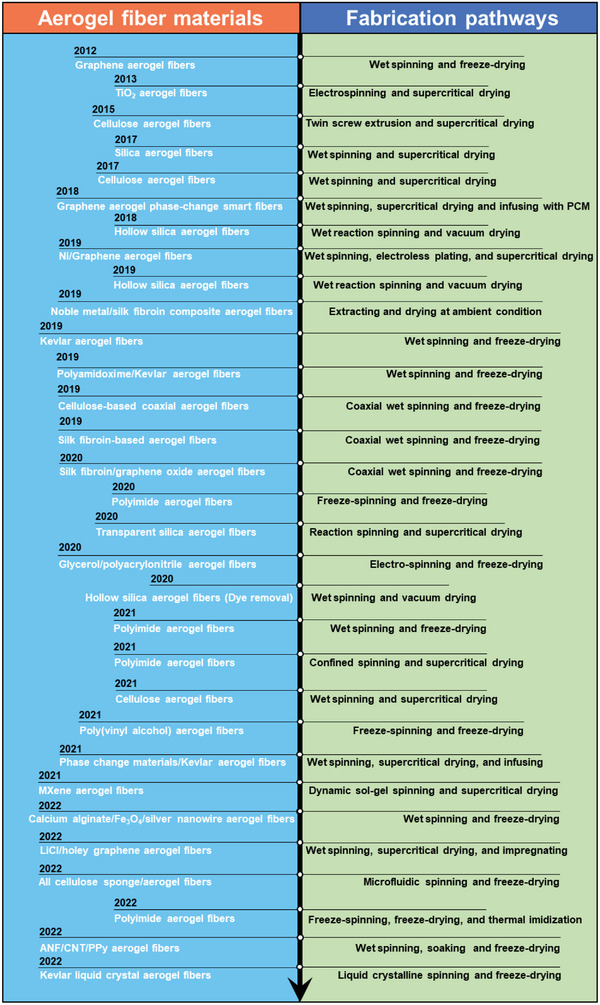
A historical timeline demonstrating the major advances in the material development and fabrication pathways of aerogel fibers.

A historical timeline of the major advances in aerogel fibers is presented in **Figure** **3**. The representative aerogel fibers and fabrication technologies have been targeted for some significant time. In 2012, Gao et al. reported the scalable wet spinning of graphene aerogel fibers from the flowing liquid crystalline graphene oxide (GO) gels. The uniformity of GO liquid crystals endows the aerogel fibers with high tensile strength and high compression modulus.^[^ ^18^ ^]^ After that, graphene‐based aerogel fibers were developed for different applications. Graphene aerogel phase‐change smart fibers were fabricated via wet spinning, supercritical drying, and infusing with phase change materials, exhibiting flexible, strong, self‐cleaning properties as well as tunable multi‐response to external stimuli (electric/optical/thermal field).^[^ ^15^ ^]^ Ni/Graphene aerogel fibers were reported by electroless plating of Ni into the wet‐spun reduced GO hydrogel fibers, possessing superior electrothermal response and EMI shielding performance with the EMI shielding effectiveness above 30 dB at the bandwidth of 12.5−20 GHz.^[^ ^37^ ^]^ Core‐sheath silk fibroin/GO aerogel fibers obtained via coaxial wet spinning and freeze‐drying, show excellent thermal insulation and infrared radiative heating function promising for personal thermal management.^[^ ^38^ ^]^ LiCl/holey graphene aerogel fibers were realized by the wet spinning of chemically etched GO, supercritical drying, and impregnating of hygroscopic salt solution, enabling integrated multifunction of highly efficient water capture, sorption‐based heating/cooling, and microwave absorption.^[^ ^9^ ^]^ In addition to graphene, MXene was also introduced to structure the aerogel fibers via dynamic sol–gel spinning followed by supercritical drying, realizing a highly oriented mesoporous structure in combination with ultrahigh electrical conductivity and electrical/optical responsiveness.^[^ ^16^ ^]^

Right after the first reported graphene aerogel fibers, TiO_2_ aerogel fibers were proposed in 2013, which were developed by electrospinning and supercritical drying in sequence.^[^ ^39^ ^]^ Besides, other types of oxide, specifically, silica aerogel fibers appeared in 2017.^[^ ^40^ ^]^ Then, Zhu et al. demonstrated hollow silica aerogel fibers obtained by wet reaction spinning and vacuum drying, showing outstanding mass transport properties for adsorption, catalysis, and sensing.^[^ ^32^, ^41^, ^42^ ^]^ In 2020, Zhang et al. reported transparent silica aerogel fibers via reaction spinning and supercritical drying, with elaborate control of optical transparency, excellent thermal insulation, and designable hydrophobicity that are suitable for wearable applications.^[^ ^22^ ^]^

Apart from carbon‐based and inorganic‐based aerogel fibers, polymer‐based aerogel fibers become more and more attractive. Starting from 2015, cellulose aerogel fibers have been developed by twin screw extrusion,^[^ ^43^ ^]^ wet spinning,^[^ ^44^ ^]^ coaxial wet spinning,^[^ ^45^ ^]^ or microfluidic spinning,^[^ ^25^ ^]^ and dried by either supercritical drying or freeze‐drying. With these approaches, homogeneous,^[^ ^43^, ^44^ ^]^ core‐sheath,^[^ ^45^ ^]^ and graded cellulose aerogel fibers^[^ ^25^ ^]^ could be achieved with high porosity, low thermal conductivity, and distinct biodegradability. In 2019, Kevlar aerogel fibers were reported by the methodology of wet spinning and freeze‐drying, rendering wide‐temperature thermal stability (≈ −196–300 °C) with various derived functionalities such as hydrophobicity, electric conductivity, and phase change behavior.^[^ ^13^ ^]^ Later, Kevlar liquid crystal aerogel fibers were developed by the same group via liquid crystalline spinning and freeze‐drying, with tailorable different building block orientations.^[^ ^35^ ^]^ Furthermore, different guest species were introduced into the Kevlar aerogel fibers, for example, with decorated polyamidoxime, they were able to extract uranium from seawater,^[^ ^46^ ^]^ and with the infusion of phase change components, they showed desirable bending stiffness that could be woven into textiles or utilized as shape memory devices.^[^ ^26^ ^]^ Since 2020, polyimide aerogel fibers have been fabricated by freeze‐spinning,^[^ ^23^, ^29^ ^]^ wet spinning,^[^ ^24^ ^]^ or confined spinning,^[^ ^21^ ^]^ and later dried by freeze‐drying or supercritical drying. They exhibited thermal insulating, fire‐retardant, and mechanically strong and flexible properties,^[^ ^21^, ^29^ ^]^ as well as phase change, electromagnetic shielding, and photothermal behavior with further functionalization.^[^ ^23^, ^24^ ^]^ In addition, other polymer aerogel fibers such as poly(vinyl alcohol) aerogel fibers,^[^ ^47^ ^]^ and composite aerogel fibers such as noble metal/silk fibroin,^[^ ^48^ ^]^ cellulose acetate/polyacrylic acid,^[^ ^49^ ^]^ glycerol/polyacrylonitrile,^[^ ^50^ ^]^ calcium alginate/Fe_3_O_4_/silver nanowire,^[^ ^28^ ^]^ and aramid nanofibers/carbon nanotube/polypyrrole (ANF/CNT/PPy)^[^ ^51^ ^]^ aerogel fibers have also been reported, which will not be discussed in detail. From the above‐mentioned fabrication pathways, confined‐spinning is a kind of batch spinning process, while all other methods are continuous spinning processes. Technically, those continuous spinning technologies can be integrated with multiple spinning nozzles, likely to scale up the final production of aerogel fibers.

### 2.2 Spinning Thermodynamics

**Figure 4:**
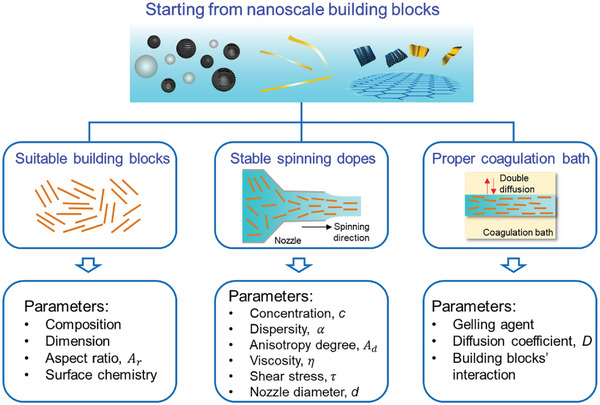
Spinning thermodynamics of aerogel fibers. Suitable building blocks, stable spinning dopes, and proper coagulation baths are three dominating factors. Porous spheres: Reproduced with permission.^[^ ^36^ ^]^ Copyright 2017, Elsevier B.V. Graphene sheets: Reproduced with permission.^[^ ^57^ ^]^ Copyright 2017, American Chemical Society. Other components in this figure are self‐produced by the authors.

Taking the nanoscale building blocks as the starting materials, diverse aerogel fibers can be constructed by the spinning technique. Three essential components should be considered to obtain the desired aerogel fibers: suitable building blocks, stable spinning dopes, and proper coagulation baths (**Figure** **4**). For the building blocks, the composition, dimension, aspect ratio, and surface chemistry are prominent factors. There is a bunch of toolboxes for the selection of nanoscale building blocks; hence, the composition can be tailored based on what kind of nanomaterials is utilized. For instance, one can apply the macromolecules (peptides, chitosan, block copolymers),^[^ ^52^ ^]^ nanoparticles (silica, TiO_2_, etc.),^[^ ^22^, ^39^, ^42^ ^]^ nanofibers (Kevlar nanofibers, cellulose nanofibers, etc.),^[^ ^13^, ^43^ ^]^ and nanoflakes (graphene, MXene, etc.)^[^ ^9^, ^15^, ^16^ ^]^ as the building blocks to structure the aerogel fibers. The dimensions of the building blocks include 0D, 1D, and 2D. The aspect ratio of the nanomaterials determines whether the building blocks could form the liquid crystal phase in the spinning process.^[^ ^53^ ^]^ The surface chemistry of the building blocks affects the interaction between the nano components and how well they can be dispersed/soluble in a certain solvent.^[^ ^53^ ^]^

For the spinning dopes, the concentration, dispersity, anisotropy degree, as well as rheological behavior including viscosity and shear stress of the dispersion should be taken into account.^[^ ^54^ ^]^ The higher the concentration, the more possible it favors to form the liquid crystalline phase during spinning.^[^ ^55^ ^]^ For instance, based on Onsager's theory, when the volume fraction of graphene sheets is above a critical value *ϕ*_c_,^[^ ^53^ ^]^

(1) $$\phi_{c}\approx 4T/W$$

such 2D sheets tend to form liquid crystals in the dispersions. In this equation, *T* and *W* stand for the monatomic thickness and lateral width of the graphene sheet, respectively. Therefore, the spinning dope with a higher anisotropy is prone to the possible formation of liquid crystals.^[^ ^53^ ^]^ In this case, Herman's factor is used to characterize the orientation of the liquid crystalline phases, which is referred to the component alignment at short and long length scales.^[^ ^54^ ^]^ The rheological behavior of the spinning dope can tell the spinnability, through which we can understand the intrinsic properties of the nanoscale constituents that they reorient during the application of shear; and therefore, ascertain how to control the microstructure of the aerogel fiber. The dynamic yield strength of the dope (i.e., the minimum force to initiate the flow through a spinneret) offers direct insight into the interaction of the nanoscale building blocks.^[^ ^54^ ^]^ Besides, it is also critical to select a suitable spinning nozzle; the nozzle diameter can not only determine the final diameter of the fiber but also enables a wide range of possibilities including those materials with a high concentration that are not accessible because of the viscosity limitations.

For the coagulation bath, a proper gelling agent should be selected because the double diffusion at the spinning dope–coagulation bath interface and the interaction between nanoscale building blocks are both important. On the one hand, the spinning dopes can be physically crosslinked and induced by temperature, pH, or salt. In this case, the spinning solute networks are often held together by electrostatic, hydrogen bonding, van der Waals, *π*–*π* stacking or hydrophobic interactions, or a combination thereof.^[^ ^56^ ^]^ On the other hand, the constitutive nanoscale building blocks can be chemically cross‐linked by using divalent cations or intermolecular disulfide bonds.^[^ ^52^, ^56^ ^]^

### 2.3 Spinning Kinetics

**Figure 5:**
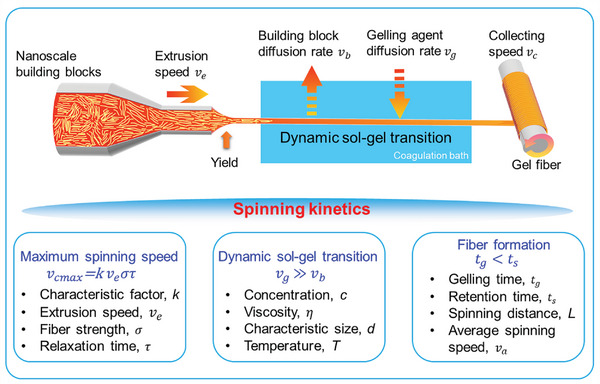
Spinning dynamics of aerogel fibers. During the dynamic sol–gel transition, the gelling agent diffusion rate competes with the building block diffusion rate to form the gel fiber.

During the spinning process, four speeds have to be considered for the spinning kinetics (**Figure** **5**), that is, extrusion speed *v*_e_, building block diffusion rate *v*_b_, gelling agent diffusion rate *v*_g_, and collecting speed *v*_c_. The spinnability of the desired dope should be related to the viscosity, velocity, and surface tension of the spinning dope,^[^ ^58^ ^]^ that is,

(2) $$S\;=\frac{\eta v_{e}}{\gamma }\;$$

where, *η*, *γ*, and *v*_e_ are the viscosity, surface tension, and extrusion speed of the spinning dope, respectively.

When spinning into the coagulation bath, the fiber undergoes a dynamic sol–gel transition, during which the gelling agent diffusion rate competes with the building block diffusion rate. The diffusion rate can be expressed as,^[^ ^59^ ^]^

(3) $$v\;=\;-\frac{kT}{6\pi \mu d}\nabla c$$

where, k is Boltzmann's constant, *T* is the temperature, µ is the viscosity of spinning dope (or coagulation bath), *v* is the building block (or gelling agent) diffusion rate, *d* is the characteristic size of building block (or gelling agent), and *c* is the concentration of building block (or gelling agent). If the gelling agent diffusion rate far outweighs the building block diffusion rate (i.e., *v*_g_ ≫ *v*_b_), the gel fiber can be successfully formed. Thus, the concentration of both nanoscale building blocks and gelling agent, the viscosity of the spinning dope and the coagulation bath, as well as the characteristic size of the building blocks and molecular size of gelling agent have to be taken into account. As for the final formation of the gel fiber, the gelling time and retention time (the ratio of spinning distance to average spinning speed) are also critical. To ensure the fiber formation, the gelling time has to be smaller than the retention time (i.e., $t_{g}=\frac{c_{g}}{dv_{g}}\;<\;t_{s}=\frac{L}{v_{a}}\;$), where, *c*_g_ and *v*_g_ represent the concentration of gelling agent and gelling agent diffusion rate, *L* is the spinning distance, and *v*_a_ is the average spinning speed.

### 2.4 Spinning Methodology

In this section, we will mainly unravel the representative spinning techniques involving either a static sol–gel transition or a dynamic sol–gel transition, including confined spinning, freeze spinning, wet spinning, reaction spinning, liquid crystalline spinning, and microfluidic spinning. For each particular technology, the mechanism, the scope, as well as the representative aerogel material will be introduced.

**Figure 6:**
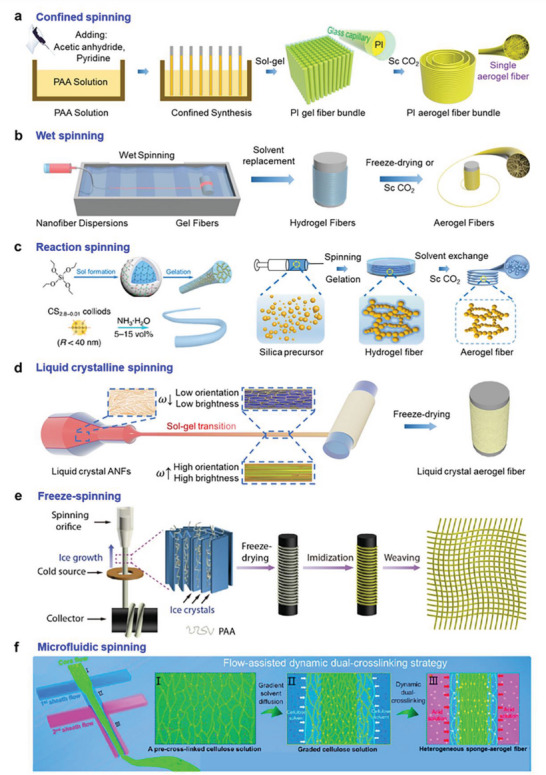
Fabrication methodologies of aerogel fibers. a) The confined spinning of polyimide aerogel fibers. Reproduced with permission.^[^ ^21^ ^]^ Copyright 2021, American Chemical Society. b) The wet spinning of Kevlar aerogel fibers. Reproduced with permission.^[^ ^13^ ^]^ Copyright 2019, American Chemical Society. c) The reaction spinning of SiO_2_ aerogel fibers. Reproduced with permission.^[^ ^22^ ^]^ Copyright 2020, American Chemical Society. d) The liquid crystalline spinning of Kevlar aerogel fibers. Reproduced with permission.^[^ ^35^ ^]^ Copyright 2022, American Chemical Society. e) The freeze‐spinning of polyimide aerogel fibers. Reproduced with permission.^[^ ^29^ ^]^ Copyright 2020, Elsevier B.V. f) The microfluidic spinning of cellulose‐graded aerogel fibers. Reproduced with permission.^[^ ^25^ ^]^ Copyright 2022, American Chemical Society.

In confined spinning, the sol–gel transition occurs in a capillary tube, where the precursor solution (e.g., polyamic acid solution) is introduced into the tubes by capillary force. This approach transforms the conventional dynamic spinning of aerogel fibers into a static sol–gel process within a confined space, resulting in a slow fiber formation in the capillary tube rather than a rapid gelling during the conventional procedure (**Figure** **6**a). Then, the aerogel fibers can be obtained after supercritical CO_2_ drying, creating fibers with diameters in the range of tens to several hundreds of micrometers. The length of the fibers via confined spinning is tightly related to the following parameters,

(4) $$L\;=\frac{2\gamma cos\theta }{\rho grsin\alpha }\;$$

where, *L* is the length of the fiber, *γ* and *ρ* represent the surface tension and density of the precursor solution, *r* is the radius of the capillary tube, *θ* is the contact angle between the precursor solution and the capillary tube, and *α* is the tilt angle of the capillary tube. This strategy is applicable to a host of different materials. Indeed, aerogel fibers comprised of organic matter (polyimide, agarose, aramid nanofiber, resorcinol formaldehyde), inorganic matter (SiO_2_, graphene, carbon), and composite matter (PI/SiO_2_, graphene/carbon nanotube, aramid nanofiber/carboxymethyl cellulose) have been successfully fabricated by confined spinning, implying a good universality of this approach.^[^ ^21^ ^]^

Distinguished from the static sol–gel transition in the confined spinning method, the wet spinning process involves the dynamic sol–gel transition to produce the aerogel fibers. On the one hand, depending on whether there is a hydrolysis–condensation reaction during the sol–gel transition, the spinning methodology can be divided into conventional wet spinning and reaction spinning. On the other hand, depending on whether liquid crystal phases are formed during the spinning, the spinning methodology can be categorized into conventional wet spinning and liquid crystalline spinning. If otherwise specifically mentioned, wet spinning in this paper refers to conventional wet spinning. In the following part, wet spinning, reaction spinning, and liquid crystalline spinning will be successively introduced.

In a wet spinning process, the spinning dope (e.g., nanofibrous Kevlar dispersion) is pressurized into the coagulation bath with a syringe pump at a certain constant speed, whereas the gel fiber is simultaneously collected in the bath. Then, the gel fibers undergo solvent replacement and freeze‐drying/supercritical drying in sequence, to form the aerogel fibers (Figure 6b).^[^ ^13^ ^]^ This technique produces aerogel fibers with diameters in hundreds of micrometers range depending on the spinning nozzle that is applied. So far, wet spinning has been conducted to fabricate a broad range of aerogel fibers, such as Kevlar,^[^ ^13^ ^]^ cellulose,^[^ ^44^, ^45^, ^60^ ^]^ graphene,^[^ ^18^ ^]^ MXene,^[^ ^16^ ^]^ and composite^[^ ^9^, ^15^ ^]^ aerogel fibers.

Reaction spinning permits rapid gelation of condensed silica colloidal particles with the help of high concentrated alkali catalyst in the coagulation bath, finalizing the formation of silica fiber rather than disassembling the colloidal particles. Similar to wet spinning, reaction spinning includes the steps of spinning and supercritical drying, and during the spinning, the silica precursor experiences the hydrolysis and condensation reaction (Figure 6c). Notably, the gelation speed is of crucial importance, neither too fast nor too slow so that the blockage of the needle or disintegration in the coagulation bath can be avoided. Hence, the nature of the condensed silica and the coagulation bath are two dominating factors: the former relies on the H_2_O/tetraethoxysilane molar ratio and HCl concentration, while the latter depends on the ammonia concentration. In principle, the success of reaction spinning is decided by the balance between the particle diffusion and chemical reaction, that is, as long as a suitable reactant meets a tunable gelation rate, it is possible to obtain the inorganic aerogel fibers. Currently, this technique has been applied to silica and TiO_2_ aerogel fibers, resulting in fibers with a surface area of 300–890 m^2^ g^−1^ and a diameter of several hundred micrometers.^[^ ^22^, ^32^, ^39^, ^42^ ^]^

In liquid crystalline spinning, the liquid crystalline spinning dope (e.g., nanofibrous Kevlar liquid crystalline dispersion) is extruded from the spinneret into a coagulation bath (e.g., water), where it undergoes a sol–gel transition and is collected simultaneously. The ratio of collection speed to extrusion speed is defined as the draft ratio. The gel fibers with various orientation degrees can be obtained via tailoring the draft ratio. Interestingly, the gel fiber with different building block orientations will show distinct brightness and color under the polarized light, enabling information encryption and decryption by utilizing the interaction between the fiber and solvent.^[^ ^35^ ^]^ Moreover, through liquid crystalline spinning, graphene aerogel fibers with superb tensile strength can be realized due to the uniform alignment of the graphene sheets inherited from the flowing dope of graphene oxide liquid crystals.^[^ ^18^ ^]^ Apart from the mechanical strength, the fibers with desirable elasticity may also be feasible, for instance, by the liquid crystalline spinning of spider silk. Through this technique, the fiber's macromolecular rods and springs can be well aligned, and the absolute size of the macromolecules as well as their distribution will contribute to the toughness of the thread.^[^ ^55^ ^]^

Freeze‐spinning refers to the technique in which the spinning dope (e.g., poly[amic acid] solution) is extruded from a syringe pump whereas the resulting hydrogel is then frozen gradually by passing through a copper ring as the cold source. By manipulating the extrusion speed and the temperature of the cold source, the fiber can be frozen at a tailorable freezing speed, determining the final structure of the fiber. As a result, the final aerogel fiber is collected by a motor and further freeze‐dried. For the poly[amic acid] fiber, a programmed thermal imidization process needs to be implemented to transform it into polyimide. The resulting polyimide aerogel fibers can then be woven into a textile for further characterization (Figure 6e). The inner porous nature of the fibers profoundly impacts their mechanical and thermal properties; diverse fibers with distinctive porous structures can be synthesized by controlling the parameters such as solution concentration, solution viscosity, extrusion speed, and freezing temperature.^[^ ^61^ ^]^ The average pore size of the aerogel fiber decreases with deducing the freezing temperature down to the liquid nitrogen point, which can be varied between −30 °C and −196 °C.^[^ ^29^ ^]^ The fibers with either well‐aligned pores or random pores can be obtained depending on the freezing temperature, which can be attributed to the freezing rate that affects the ice nucleation and growth.^[^ ^61^ ^]^

Microfluidic spinning utilizes the microfluidic chip to create designable flows in the spinning of fibers, enabling aerogel fibers with graded porous structure or complicated chemical composition.^[^ ^62^, ^63^, ^64^, ^65^ ^]^ For instance, this technique has been employed to produce cellulose‐based graded aerogel fibers. In the microfluidic chip, a chemically crosslinked cellulose solution is taken as the core flow, that is, spinning dope, which travels through two sheath flow channels, containing a diffusion solvent as the first sheath flow and a physical crosslinking solvent as the second sheath flow. This process results in graded sponge–aerogel composite fibers with a dense aerogel inner layer and a porous sponge outer layer (Figure 6f). By regulating the flow process in the microfluidic chip, in combination with the computational fluid dynamics simulation, the aerogel fibers with heterogeneous structure, controllable spinnability, and desirable mechanical and thermal properties can be customized. The fiber diameter can be obtained in the range of ≈32–163 µm, with the sheath thickness of ≈2–7.5 µm and a maximum length of up to 56 m. In addition, the rheological properties of the spinning dope during the microfluidic flow largely rely on the solution concentration, flow rate, and the interaction between the dope and the confined geometry of the channels.^[^ ^63^ ^]^ On the basis of a comprehensive understanding of hydromechanics and reaction dynamics, this flow‐assisted strategy will be of huge significance for constructing biomass fibers and textiles with complex structures, greatly directing green chemistry.^[^ ^25^ ^]^ Apart from the cellulose‐based aerogel fibers, other materials are likely to be utilized in microfluidic spinning such as alginate,^[^ ^62^ ^]^ graphene,^[^ ^63^, ^65^ ^]^ carbon nanotubes,^[^ ^64^ ^]^ or their composites.

Among the current spinning methodologies of aerogel fibers, each method has its pros and cons. Confined spinning can be applied to a variety of materials, and it is advantageous to understand the static sol–gel transition for different material systems. From the viewpoint of spinning efficiency, confined spinning tends to be a batch process; and thus, the length of fiber would be limited. Wet spinning, reaction spinning, liquid crystalline spinning, freeze‐spinning, and microfluidic spinning can be utilized to fabricate continuous aerogel fibers with a large amount. In addition, those approaches are easier to be scaled up by integrating multiple spinning nozzles or in a parallel manner. Wet spinning applies to a broad range of spinning dopes with low to high concentrations, while liquid crystalline spinning is restricted to those with a certain critical high concentration when the spinning dope falls into the liquid crystalline phase. Reaction spinning is focused on the materials that would undergo hydrolysis and condensation reactions. Microfluidic spinning can be proposed to synthesize aerogel fibers with variant compositions in a single fiber along the spinning process.

### 2.5 Drying Methodologies

After the gel formation, drying is a necessary step to obtain the final aerogel fibers. It is a major challenge to remove the liquid solvent from the gel while avoiding the shrinkage or cracking of the established network structure because the delicate nanostructure of the wet gel may not be able to withstand the large capillary force during drying owing to the surface tension at the liquid–gas interface.^[^ ^56^, ^66^ ^]^ Currently, three methods are employed for drying aerogel fibers, which are designed to eliminate or minimize the capillary forces caused by the surface tension effect. They are i) ambient pressure drying, ii) freeze drying, and iii) supercritical drying.

Ambient pressure drying is assigned to dry the wet gel at the condition of ambient pressure. It usually requires multiple steps of solvent exchange to relieve the capillary force or improve the ability of the nanostructure to conquer those forces.^[^ ^66^ ^]^ Hence, this drying technique largely depends on the surface modification of the internal gel surface, for example, silylation before performing the actual drying procedure. The advantage of ambient pressure drying lies in the low cost, compared with both freeze‐drying and supercritical drying.^[^ ^67^ ^]^

Freeze‐drying is a fascinating method that makes a wet gel into a cryogel and removes the solvent via sublimation. Basically, the ice crystals that form during freeze‐drying may result in aerogel fibers with more macropores as compared with supercritical drying.^[^ ^13^, ^56^ ^]^ This drawback may also bring about some unique features such as layered structure with better thermal property via unidirectional freezing.^[^ ^61^, ^68^ ^]^

Supercritical drying method is by far the most efficient way to obtain aerogel fibers with minimal structure collapse. Instead of water that involves in the solvent exchange in freeze‐drying, it requires alcohol that is miscible with supercritical CO_2_. Based on the microstructure of aerogel fibers by both freeze‐drying and supercritical drying, the samples prepared by the latter method preserve better porous structures^[^ ^13^ ^]^ because the supercritical method can avoid the liquid/gas boundary line via bringing the solvent above the supercritical point.^[^ ^66^, ^69^ ^]^ In the supercritical state, there is no liquid/gas interface and thus no capillary force caused by the meniscus receding.

## 3 Material Family Maps of Aerogel Fibers

**Figure 7:**
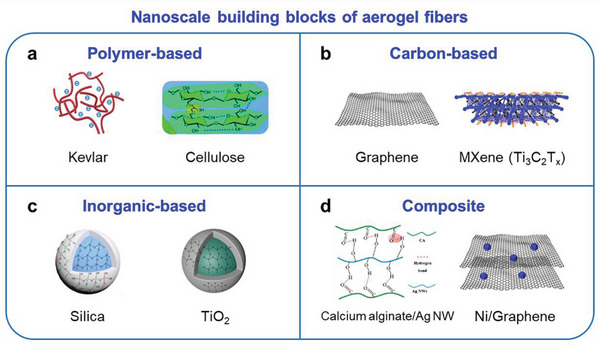
Material family maps of aerogel fibers with nanoscale building blocks as the starting materials. a) Nanoscale building blocks for polymer‐based aerogel fibers. Image of Kevlar: Reproduced with permission.^[^ ^26^ ^]^ Copyright 2021, American Chemical Society. Image of Cellulose: Reproduced with permission.^[^ ^25^ ^]^ Copyright 2022, American Chemical Society. b) Nanoscale building blocks for carbon‐based aerogel fibers. Image of graphene: Reproduced with permission.^[^ ^9^ ^]^ Copyright 2022, Springer Nature. Image of MXene: Reproduced with permission.^[^ ^16^ ^]^ Copyright 2021, Wiley‐VCH. c) Nanoscale building blocks for inorganic‐based aerogel fibers. Images of silica and TiO_2_: Reproduced with permission.^[^ ^22^ ^]^ Copyright 2020, American Chemical Society. d) Nanoscale building blocks for composite aerogel fibers. The image of calcium alginate/Ag nanowires (Ag NW): Reproduced with permission.^[^ ^28^ ^]^ Copyright 2022, American Chemical Society. The image of Ni/Graphene: Reproduced with permission.^[^ ^37^ ^]^ Copyright 2019, American Chemical Society.

With the strategy of assembling from nanoscale building blocks, there is almost no limitation for the material design of aerogel fibers. Generally, the materials can be cataloged as polymer‐based, carbon‐based, inorganic‐based, and composite aerogel fibers (**Figure** **7**). For each type of material, typical examples will be provided, including the source of the material, the preparation, and the mechanism of how to disperse these nanomaterials as the building blocks.

Polymer‐based aerogel fibers include Kevlar, cellulose, polyimide, and poly(vinyl alcohol). Kevlar nanofiber dispersions can be produced by dissolving the macro form of the well‐known aramid polymer Kevlar threads, with distinct high‐aspect ratio features in diameter of ≈3–30 nm and 30 µm in length. The negatively charged Kevlar nanofibers are uniformly dispersed in dimethyl sulfoxide (DMSO) solution by controlled deprotonation with KOH; so that, they can be exploited as the new nanoscale building blocks for strong aerogel materials.^[^ ^13^, ^26^, ^70^ ^]^ Cellulose, an abundant natural polysaccharide, mainly comes from wood, plants, bacteria, tunicate, and algae.^[^ ^71^, ^72^ ^]^ The dissolution of cellulose to make spinning dopes can transform its hydrogen pattern in crystalline structure from cellulose I into cellulose II, where cellulose I has a parallel orientation and cellulose II has an antiparallel one.^[^ ^73^, ^74^ ^]^ Due to the enormous amounts of intra‐ and inter‐hydrogen bonds in cellulose chains, cellulose can merely be dissolved by specific solvents such as tetra butyl ammonium fluoride/dimethyl sulfoxide, ionic liquids, metal‐complex solutions, *N*‐methylmorpholine‐*N*‐oxide, LiCl/*N*,*N*‐dimethylacetamide, organic solutions, alkali/urea solutions, and molten inorganic salt hydrates.^[^ ^25^, ^45^, ^73^ ^]^

The carbon‐based aerogel fibers contain graphene, MXene, carbon, and so on. Graphene is a well‐known 2D material with carbon atoms bonded in a hexagonal lattice, showing excellent thermal and electric conductivity. Taking graphene as the starting material for aerogel fibers, a dispersion of graphene oxide in an aqueous solution should be well‐prepared from the exfoliation of natural graphite. The GO sheets bear a high aspect ratio, that is, atomic‐scale thickness and micrometer‐scale width, which profoundly determines the spontaneous formation of lyotropic liquid crystals with increasing GO concentrations.^[^ ^18^ ^]^ The typical liquid crystalline behavior of GO sheets can form an aligned orientation, which can be further assembled in order to form the macroscopic fiber by the shear flow of wet spinning.^[^ ^9^, ^15^, ^75^ ^]^ MXene is a huge family of 2D metal carbides and nitrides, which consists of two or more layers of transition metal atoms intervened by carbon and/or nitrogen atoms in a honeycomb 2D lattice. The general formula of MXene is denoted as *M_n_*_+1_ *X_n_T_x_*, where *M*, *X*, and *T*, respectively stand for the transition metal, carbon/nitrogen, and surface terminations (e.g., —F, =O, or —OH), with *n* varying from 1 to 4.^[^ ^76^, ^77^ ^]^ MXene has shown outstanding and tailorable electrical, chemical, optical, and mechanical properties.^[^ ^78^ ^]^ MXene spinning dope can be produced through a top–down approach, selectively removing A‐layer atoms (e.g., Al, Si, Ga) from the MAX phases and leaving behind MX layers. Ti_3_C_2_T*_x_* MXene dispersion has been synthesized by the minimal delamination from the Ti_3_AlC_2_ MAX phase, which shows a typical shear thinning behavior that is promising for the subsequent dynamic sol–gel spinning.^[^ ^16^ ^]^ With ≈50 MXenes reported so far, in combination with a deeper understanding of the precursor structure and stoichiometry, this is a growing library of 2D materials that can be considered as building blocks for aerogel fibers.^[^ ^76^ ^]^

Examples of inorganic aerogel fibers can be drawn from SiO_2_ and TiO_2_. For silica aerogel fiber, the precursor tetraethoxysilane (TEOS) is hydrolyzed in the ethanol/acid mixed solution, which will then be converted into a condensed silica solution. The TEOS species undergo a hydrolysis reaction to form reactive silanol groups, which will then undergo condensation, resulting in Si—O—Si bridging bonds. First, a sol of condensed silica colloids is rapidly formed; and then, these aggregate into clusters with further condensation, finally forming a gel.^[^ ^66^ ^]^ To structure the aerogel fiber, the fast gelation time is controlled by modulating the H_2_O/TEOS molar ratio and the ammonia concentration in the coagulation bath. With differently sized silica colloids as the building blocks, the transparency of the resultant aerogel fibers can be well tailored. A similar methodology is also applicable to TiO_2_ aerogel fiber, which is formed by taking tetrabutyl titanate and ethanol as the spinning dope and acetic acid as the hydrolysis inhibitor in a coagulation bath.^[^ ^22^ ^]^

For the composite aerogel fibers, a variety of hybrid materials has been developed, for example, calcium alginate/Fe_3_O_4_ nanoparticles/Ag nanowires (CA/Fe_3_O_4_/Ag NWs),^[^ ^28^ ^]^ Ni/graphene,^[^ ^37^ ^]^ silk fibroin/graphene oxide,^[^ ^38^ ^]^ polyamidoxime/aramid nanofiber (PAO/ANF),^[^ ^46^ ^]^ and aramid nanofibers/carbon nanotube/polypyrrole (ANF/CNT/PPy).^[^ ^51^ ^]^ These hybrid structures can be realized by either an in situ approach or a post‐treatment such as coating the guest component. Calcium alginate/Fe_3_O_4_ nanoparticles/Ag nanowires aerogel fibers were fabricated through wet spinning, freeze‐drying, spray‐coating, and weaving processes. The mixture of sodium alginate and Fe_3_O_4_ nanoparticles was wet‐spun into a CaCl_2_ coagulation bath, forming Ca^2+^ cross‐linked alginate hydrogel fiber. After the solvent exchange and freeze drying, ammonium polyphosphate (APP) and Ag NWs were alternately sprayed onto the fiber. The carbonyl group (C=O) of poly(vinylpyrrodidone) on the Ag NWs interacted with the hydroxyl group (—OH) of CA through hydrogen bonding, ensuring interface adhesion ability. The CA/Fe_3_O_4_/Ag NWs fibers were further assembled into fire alarm electronic textiles for firefighting protective clothing.^[^ ^28^ ^]^ Ni/graphene aerogel fibers could be prepared by electroless plating of Ni nanoparticles onto the wet‐spun graphene hydrogel fibers, leading to the homogeneous decoration of polycrystalline Ni on the surface of graphene sheets and possessing excellent EMI shielding behavior.^[^ ^37^ ^]^ Silk fibroin/graphene oxide (SF/GO) aerogel fibers were obtained via the coaxial wet spinning process, with SF/GO as the core layer and cellulose acetate/poly(acrylic acid) (CA/PAA) as the sheath layer, demonstrating superb thermal insulation and infrared radiative heating performance.^[^ ^38^ ^]^ Polyamidoxime/aramid nanofibers (PAO/ANF) aerogel fibers were produced via wet spinning the aramid nanofibers into a PAO‐contained coagulation bath for in situ gelation of ANF with PAO, exhibiting selective uranium extraction in the presence of competing ions.^[^ ^46^ ^]^ ANF/CNT/PPy aerogel fibers were developed by the wet spinning of the ANF/CNT mixture while coating the PPy layer afterward. The resultant aerogel fibers showed high conductivity and tensile strength with low density, which could be exploited as motion sensors for human health detection and management.^[^ ^51^ ^]^

Moreover, a variety of other nanomaterials can be anticipated as the building blocks for aerogel fibers, for instance, metal chalcogenide nanoparticles,^[^ ^79^ ^]^ piezoelectric nanofibers,^[^ ^80^ ^]^ semiconducting nanomaterials,^[^ ^81^ ^]^ and polyethylene nanofibers.^[^ ^82^ ^]^ These are potential newcomers that are likely to enable the acoustic function,^[^ ^80^ ^]^ optical communications,^[^ ^81^ ^]^ radiative cooling,^[^ ^82^ ^]^ and beyond. In addition to the source of the material, the preparation, and the mechanism of dispersing the nano building blocks mentioned above, the cost, biodegradability and recyclability should also be major concerns for the material selections. Specifically, natural materials are cost‐effective, biocompatible, biodegradable, and recyclable. For instance, cellulose, lignin, cotton, chitosan, chitin, polysaccharides, starch, alginate, pectin and DNA molecules can be utilized as the building blocks for aerogel fibers.^[^ ^20^, ^56^, ^83^, ^84^, ^85^, ^86^, ^87^ ^]^ In addition, synthetic polymers such as polyglycolic acid (PGA), polylactic acid (PLA), polycaprolactone (PCL), polybutylene succinate (PBA), polyethylene adipate (PEA), and poly p‐dioxanone (PDS) are also biodegradable and recyclable.^[^ ^88^ ^]^

## 4 Fascinating Properties

Owing to the unique porous structure with ultrahigh porosity, large specific surface area, ultralow thermal conductivity, and tunable transparency, diverse aerogel fibers can be obtained with tailored mechanical, thermal, sorptive, optical, and fire‐retardant properties. For each property, the related important parameters that will guide to a better performance will be particularly discussed. The outstanding questions concerning the structure, fabrication, and properties cannot be merely addressed by the experimental study, but will require the help from computational approaches with further efforts.

### 4.1 Mechanical Properties

Most of the time, aerogel is featured with brittleness; however, aerogel fibers provide an alternative route to better flexibility, machinability, and mechanical adaptivity. Those aspects include but are not limited to tensile strength, bending stiffness, elasticity, and torsional strength. Aerogel fibers with a broad range of materials can be designed and enable the capability of bending, knotting, and weaving into large areas of textile.

**Figure 8:**
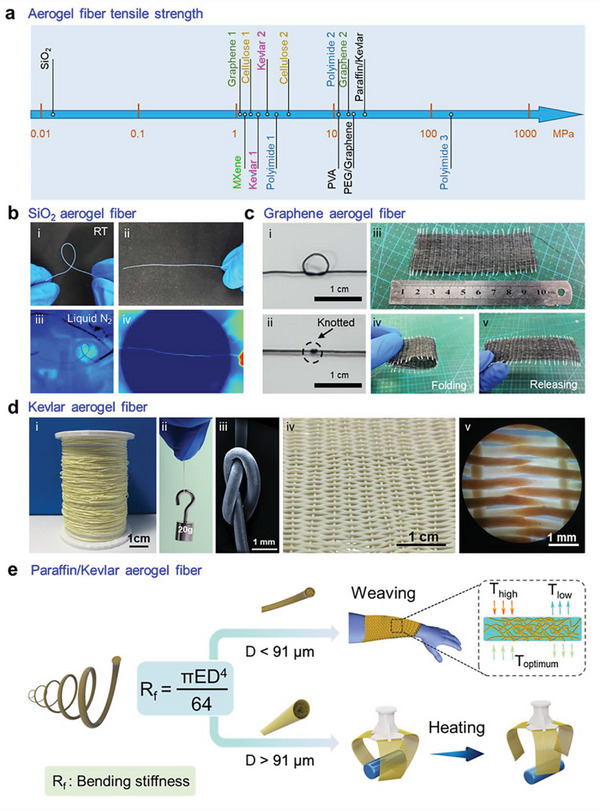
Mechanical properties of aerogel fibers. a) The tensile strength of a series of aerogel fibers. b) Silica aerogel fibers that can bend and recover at room and liquid nitrogen temperatures. Reproduced with permission.^[^ ^22^ ^]^ Copyright 2020, American Chemical Society. c) Hygroscopic holey graphene aerogel fibers that can be knotted and woven into neat textiles. Reproduced with permission.^[^ ^9^ ^]^ Copyright 2022, Springer Nature. d) Kevlar aerogel fibers with mechanical robustness including knotting and weaving. Reproduced with permission.^[^ ^13^ ^]^ Copyright 2019, American Chemical Society. e) Bending stiffness derived strategy of paraffin/Kevlar aerogel fibers toward different applications. Reproduced with permission.^[^ ^26^ ^]^ Copyright 2021, American Chemical Society.

Depending on the material nature and interactions between the nanoscale building blocks, the constructed aerogel fibers exhibit desired mechanical properties, with tensile strength ranging from tens of kilopascals to hundreds of megapascals. A transparent silica aerogel fiber has a tensile strength of 250 kPa (**Figure** **8**a), which is higher than the opaque one (150 kPa). These silica aerogel fibers also show good flexibility in a broad temperature range of −200 °C to –600 °C. A fiber with a length above 2 cm can be bent to 360° and return to the original straight shape after relaxation, at room temperature (Figure 8b‐i,ii), liquid nitrogen temperature (−200 °C, Figure 8b‐iii‐iv), or high temperature up to 600 °C.^[^ ^22^ ^]^ The hygroscopic holey graphene fibers possess a tensile strength of 1.05 MPa (Figure 8a) and can be bent (bending stiffness of 3.08 × 10^−9^ N m^2^), knotted, and woven into textiles (Figure 8c).^[^ ^9^ ^]^ After the infusion of phase change materials (PCM), the tensile strength of PCM/graphene composite aerogel fibers can be up to 12.7 MPa, where polyethylene glycol as a matrix in between the graphene nanolaminates contributes to raising the strength of the fiber.^[^ ^15^ ^]^ Kevlar aerogel fiber obtained from the 2 wt% nanofibrous dispersion shows a tensile strength of 3.3 MPa, and a single fiber with ≈300 µm in diameter can resist a load of 20 g. In the meantime, Kevlar aerogel fibers exhibit outstanding flexibility to be knotted and easily woven into textiles, eliminating any damage during the weaving process (Figure 8d).^[^ ^13^ ^]^ Imbuing the hydrophobic Kevlar aerogel fibers with functional guests such as paraffin, the composite fibers attain satisfactory mechanical properties with a tensile strength close to 30 MPa. Notably, based on the bending stiffness‐directed strategy, paraffin/Kevlar aerogel fibers with designable diameters can be derived for different applications. Those with a lower bending stiffness (fiber diameter <91 µm) than a critical value (1.22 × 10^−9^ N·m^2^) can be utilized for smart temperature‐regulating textiles, whereas those with the bending stiffness (fiber diameter >91 µm) above the threshold are applied for shape memory grips (Figure 8e).^[^ ^26^ ^]^

Despite current development, achieving aerogel fibers with higher mechanical properties needs more effort. Taking the composite nanofibers (e.g., aramid nanofibers and polyvinyl alcohol) as building blocks may be a good chance because the interactions between the nanoscale constituents can construct assembled networks with high nodal connectivity and strong crosslinking between the composite fibrils. These hyper‐connective features at fibrillar joints may result in an improvement of the macroscopic mechanical properties of the fibers.^[^ ^89^ ^]^ Besides, mechanically interlocked molecules are also likely to contribute to mechanical robustness. Due to the inherent host–guest interaction, aerogel fibers constituted of these locked molecular skeletons may achieve a combination of superior mechanical strength, responsiveness, and multiple functionality.^[^ ^90^ ^]^ Some recent works have tried to tackle the brittleness of bulk aerogels, achieving high deformability/compressibility.^[^ ^3^, ^91^ ^]^ These macroscopic aerogels with superior mechanical properties also shed a light on constructing aerogel fibers with improved properties.

### 4.2 Thermal Properties

Thermal insulation is by far the most prominent feature of aerogel materials due to the extremely low thermal conductivity (as low as 0.015 Wm^−1^ K^−1^ for silica aerogels). Aerogel fibers retain the thermal insulation capability along with their flexibility, enabling easy fabrication of textiles with complex geometries for personal thermal management. Heat transfer in the aerogel fiber obeys the following three mechanisms: heat conduction via the solid backbone material, heat transfer in the gas phase confined in the open‐porous aerogel structure, and thermal radiation.^[^ ^66^ ^]^ For the macroscopic form of the aerogel fibers, thermal properties are tightly related to the diameter of the fiber, the internal structure of the fiber, and the thickness of the final product (textile/aggregates).

**Figure 9:**
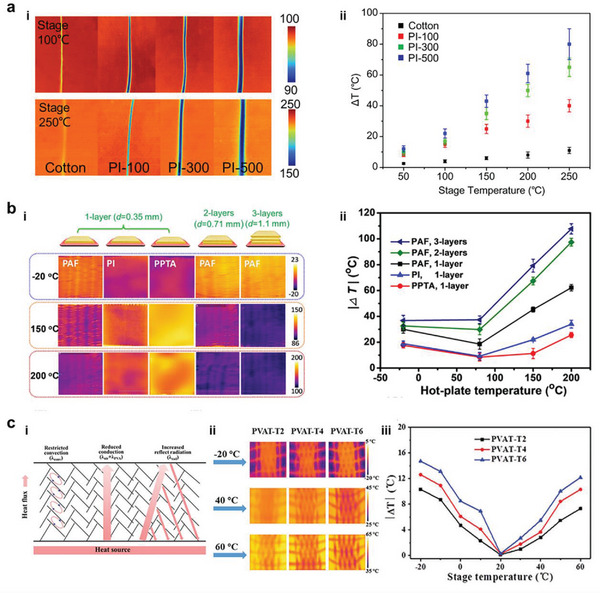
Thermal properties of aerogel fibers. a‐i) Infrared images of polyimide single aerogel fibers with variant diameters on the hot stage with temperatures of 100 °C and 250 °C, and a‐ii) the temperature difference between the fiber surface and hot stage. Reproduced with permission.^[^ ^21^ ^]^ Copyright 2021, American Chemical Society. b) Infrared images of polyimide aerogel fabrics and commercially available fabrics, and the temperature difference between the fabric surface and hot stage. Reproduced with permission.^[^ ^24^ ^]^ Copyright 2021, American Chemical Society. c) Poly(vinyl alcohol) aerogel fibers with dendrite‐like pore morphology and their thermal performance with different layers of fabrics. Reproduced with permission.^[^ ^47^ ^]^ Copyright 2021, Wiley‐VCH.

The polyimide aerogel fiber with low thermal conductivity and high porosity shows exceptional thermal insulation performance. This could be evidenced by the infrared images of polyimide aerogel fibers with different diameters. Compared with cotton fibers, polyimide aerogel fibers exhibit better thermal insulation property, and those with larger diameters display higher temperature deviation from stage temperatures. Importantly, these features become more apparent with increasing the stage temperature (**Figure** **9**a).^[^ ^21^ ^]^ When woven into fabrics, at a relatively low temperature (20 °C), polyimide aerogel fabric shows more evident thermal insulation than commercial polyimide and poly(p‐phenylene terephthalamide) (PPTA) fabrics with the same thickness. At an elevated temperature, the thermal insulation performance of polyimide aerogel fabric is even more prominent. Furthermore, the fabrics with multiple layers indicate better thermal insulation properties. The temperature difference can further tell the outstanding thermal insulation behavior, especially, the three‐layer polyimide aerogel fabric shows the absolute temperature difference of 110 °C at the hot stage of 200 °C, outperforming the single‐layered scenarios (Figure 9b).^[^ ^24^ ^]^

The thermal conductivity of an aerogel fiber can be determined by,^[^ ^47^ ^]^

(5) $$\lambda_{f}=\lambda_{\mathrm{conv}}\;+\lambda_{\mathrm{air}}+\lambda_{\mathrm{solid}}+\lambda_{\mathrm{rad}}$$

where, *λ*_conv_ and *λ*_rad_ represent the thermal convection and thermal radiation of the aerogel fiber, and *λ*_air_ and *λ*_solid_ refer to the thermal conductivity of gas and solid material in the fiber, respectively. Poly(vinyl alcohol) (PVA) aerogel fibers with well‐aligned dendrite‐like porous structures can restrict the thermal radiation of the fiber, owing to the abundant reflective faces. In addition, numbers of cells are separated by the branches of the dendrite‐like pore network, profoundly impeding the thermal convection of the fiber because the air can be blocked in the individual cells. The high porosity of the aerogel fiber will lead to slight thermal conduction. PVA aerogel fabrics with different layers are placed on the heating sources, and a huge temperature difference is realized by merely one thin layer, indicating superb thermal insulation. Similarly, the thermal insulation performance is improved with more layers of textiles (Figure 9c).^[^ ^47^ ^]^

The direct thermal conductivity characterization of a single aerogel fiber is challenging; simulation (e.g., COMSOL Multiphysics) is an alternative way to deepen the understanding of heat transfer happening in the aerogel fiber. The aerogel fiber can be simplified as the intersecting square rod structure in which one cell is taken for the analysis. The thermal conductivity of the cell can be expressed as:^[^ ^92^ ^]^

(6) $$k\;=\left[\begin{array}{ccc} k_{xx} & k_{xy} & k_{xz}\\ k_{yx} & k_{yy} & k_{yz}\\ k_{zx} & k_{zy} & k_{zz} \end{array}\right]\;$$

Assuming the aerogel fiber as a homogeneous isotropic material, *k_xx_* = *k_yy_* = *k_zz_*, and the nondiagonal terms should be zero. Thus, the thermal conductivity of only one direction needs to be calculated. Based on Fourier's law of heat transfer, the thermal conductivity along *x* direction can be determined as,^[^ ^92^ ^]^

(7) $$k_{xx}=\frac{\left\langle Q_{x}\right\rangle L_{x}}{\Delta T}\;$$

where, *Q_x_* and *L_x_* stand for the heat flux through the *x* direction and the cell length in the *x* direction, respectively. Δ*T* represents the temperature difference. 〈 〉 refers to the average operator on the boundary where temperature constrain is set. Accordingly, the thermal conductivity of aerogel fibers increases with increasing temperatures, while decreasing with the increase of the pore diameter and the decrease of the skeleton. Therefore, it is feasible to reduce the diameter of nanofibers to better the thermal insulation property.^[^ ^13^ ^]^

### 4.3 Sorptive Properties

The controllable pore size, high porosity, and high specific pore volume of aerogel fibers enable them to be ideal candidates for adsorbing or extracting chemical compounds, for instance, water adsorption, ion adsorption, and pollutant adsorption. Here, we will particularly describe the water adsorption mechanism of the holey graphene aerogel fibers that are decorated with hygroscopic salts, the Kevlar aerogel fibers that are modified with polyamidoxime for uranium extraction, and the silica aerogel fibers with the hollow structure for dye pollutant removal.

**Figure 10:**
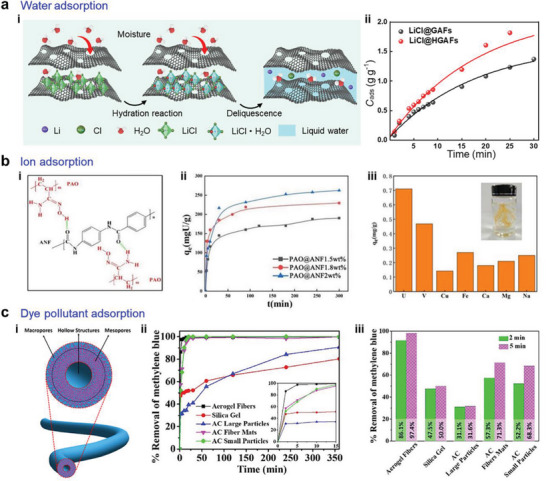
Sorption properties of aerogel fibers. a) Water adsorption of hygroscopic holey graphene aerogel fibers. Reproduced with permission.^[^ ^9^ ^]^ Copyright 2022, Springer Nature. b) Ion adsorption of Kevlar aerogel fibers during uranium extraction. Reproduced with permission.^[^ ^46^ ^]^ Copyright 2019, MDPI. c) Dye pollutant adsorption of silica aerogel fibers. Reproduced with permission.^[^ ^32^ ^]^ Copyright 2019, Elsevier B.V.

The holey graphene sheets in the aerogel fibers provide abundant surface area and binding sites for water capture and sufficient pathways for water transport by virtue of etched nanopores. The water capture mainly follows three steps: i) the chemisorption of water by LiCl on the fiber to form LiCl·H_2_O, ii) the deliquescence of LiCl·H_2_O to LiCl solution, and iii) the continuing water sorption by LiCl solution to a more diluted solution (**Figure** **10**a‐i). The sorption process takes advantage of both the high water uptake kinetic of solid adsorbents and the high water uptake capacity of liquid adsorbents. During the initial 30 min interval, LiCl@HGAFs exhibit faster water uptake kinetics compared with LiCl@GAFs, originating from the plenty of diffusion pathways through the etched nanopores (Figure 10a‐ii). As the sorption time extends to 350 min, the water capture capacity of the two materials will approach, due to the deliquescence of LiCl∙H_2_O into the aqueous LiCl solution inside the fiber so that liquid sorption will continue to dominate the sorption process.^[^ ^9^ ^]^

By anchoring polyamidoxime (PAO) on the skeleton of Kevlar nanofiber, PAO/Kevlar aerogel fibers are successfully realized with the function of uranium extraction, where PAO acts with Kevlar nanofiber through hydrogen bonds in the formation of the gel fiber (Figure 10b‐i). The uranium adsorption occurs in two processes: fast adsorption in the initial 10 min and slow uptake until the sorption saturation. The adsorption capacity is tightly related to the concentration of Kevlar nanofibers, that is, a higher nanofiber concentration leads to a higher surface area of the aerogel fiber; and therefore, a higher uptake capacity (Figure 10b‐ii). The highest uranium adsorption capacity of aerogel fibers is 262.5 mg g^−1^, surpassing that of amidoxime‐grafted active carbon fibers and covalent organic framework modified platforms. In the presence of the coexisting ions, the PAO/Kevlar aerogel fibers possess the highest sorption capacity for uranium, illustrating high selectivity for UO_2_^2+^ due to its complexation with amidoxime groups (Figure 10b‐iii).^[^ ^46^ ^]^

Silica aerogel fibers with hollow and hierarchical structures were achieved via wet reaction spinning, showing a high adsorption rate for dye pollutants. The high adsorption performance is attributed to the superhigh specific surface area and interconnected pores. The formation of the hierarchical structure is related to three primary processes: i) The surface of water glass can be quickly converted into solid orthosilicic acid, leading to an epidermal layer with microporous size and high modulus. ii) Before the reaction with sulfuric acid, the fiber center of the water glass will diffuse outward with the solvent and gather around the interior surface of the epidermal layer, resulting in a hollow structure in the central zone. iii) During the aging process, the unreacted water glass near the inner surface of the epidermal layer reacts with sulfuric acid, forming the secondary network with much denser and smaller pores (mesopores) (Figure 10c‐i). The adsorption rates of methylene blue (MB) are rapid in the first few minutes, with the MB removal rates by silica aerogel fibers of 86.1% and 97.4%, respectively in 2 and 5 min. These performances are at least 25% higher than commercial sorbents such as silica gel, activated carbon particles, and activated carbon fiber mats (Figure 10c‐ii‐iii).^[^ ^32^ ^]^

### 4.4 Optical Properties

**Figure 11:**
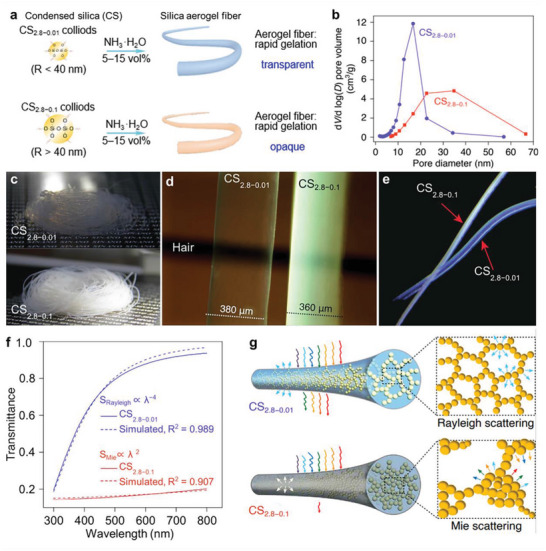
Optical properties of silica aerogel fibers. a) The schematic of condensed silica and its formation into aerogel fibers. b) Pore size distribution of CS_2.8‐0.01_ and CS_2.8‐0.1_ aerogel fibers. c) Digital photos illustrating the CS_2.8‐0.01_ transparent aerogel fibers and CS_2.8‐0.1_ opaque aerogel fibers. d) Optical image of a CS_2.8‐0.01_ aerogel fiber and a CS_2.8‐0.1_ aerogel fiber on a hair. e) Optical photo of the light blue CS_2.8‐0.01_ aerogel fiber and white CS_2.8‐0.1_ aerogel fiber. f) The transmittance of the CS_2.8‐0.01_ and CS_2.8‐0.1_ aerogel films with the thickness of 2 mm. g) Schematics of the silica aerogel fibers based on Rayleigh and Mie scattering. (a–g) Reproduced with permission.^[^ ^22^ ^]^ Copyright 2020, American Chemical Society.

Aerogel fibers can be see‐through via tuning the transparency. For example, transparent silica aerogel fibers are realized by a reaction spinning process when the condensed silica colloidal particle size can be adjusted during tetraethoxysilane (TEOS) hydrolysis. The colloidal size is largely related to the concentration of HCl. The particle size ranges from 24 to 50 nm within the HCl concentration range of ≈0.01–0.1 m. Further reducing the HCl concentration will lead to a failure for condensed silica. Further increasing the HCl concentration would cause precipitation during turbulence. For the aerogel fiber with a colloidal size smaller than 40 nm (denoted as CS_2.8‐0.01_), the light scattering is dominated by Rayleigh scattering. When the colloidal size is much larger (denoted as CS_2.8‐0.1_), a Mie scattering dominates the light scattering, making the aerogel fiber opaque (**Figure** **11**a,b).

Silica aerogel fibers produced from CS_2.8‐0.01_ and CS_2.8‐0.1_ show variant appearances, that is, the former is transparent whereas the latter is opaque (Figure 11c–e). The transmittance of the transparent and opaque aerogel fibers is determined as 92% and 20%, respectively. To further understand the light transmission, the films made of the two types of colloids are fabricated. For the CS_2.8‐0.01_ film, the light transmittance increases abruptly from 20% to 80% in the range of 300–500 nm, whereas it is below 10% in the range of ≈200–850 nm for the CS_2.8‐0.1_ film. For the CS_2.8‐0.01_ film with a colloidal size smaller than 40 nm, the transmittance of silica aerogel fiber obeys to Rayleigh–Gans theory,^[^ ^93^ ^]^

(8) $$S_{\mathrm{Rayleigh}}=\frac{I}{I_{0}}\;=\;\exp\left[-32\pi^{4}\frac{\rho_{\mathrm{fiber}}}{\rho_{{\mathrm{SiO}}_{2}}}\frac{d^{3}bc}{\lambda^{4}}{\left(\frac{m^{2}-1}{m^{2}+2}\right)}^{2}\right]$$

where, *ρ*_fiber_ and $\rho_{{\mathrm{SiO}}_{2}}$ represent the density of a silica aerogel fiber and the density of bulk silica, respectively; *d*, *b*, and *c* stand for the size of silica particles, volume fraction of the silica aerogel, and film thickness, respectively, *λ* refers to the wavelength of the incident light, and *m* is the relative refractive index (the ratio of the refractive index of silica and that of air). For the CS_2.8‐0.1_ film with a much larger particle size, a Mie scattering is considered for the transmittance,^[^ ^94^ ^]^

(9) $$S_{\mathrm{Mie}}=\frac{I}{I_{0}}\;=\frac{\lambda^{2}}{4\pi^{2}r^{2}}\;\left(i_{1}sin^{2}\varphi +i_{2}cos^{2}\varphi \right)\propto \lambda^{2}$$

where, *λ* is the wavelength of the incident light, *r* represents the distance between the particle and the observation point, *φ* is the angle between the vibration surface and the scattering surface, and *i*_1_ and *i*_2_ stand for the Mie scattering intensity functions related to the incident light wavelength, particle size, relative refractive index, and the scattering angle, respectively (Figure 11f,g).^[^ ^22^ ^]^

Moreover, through the elaborate size tunability of silica building blocks, silica aerogel fibers or those woven into fabrics might be possible to be used for laser‐driven lighting. Based on the previous research by Zhang et al.,^[^ ^30^ ^]^ the silica aerogel with spherical building blocks of ≈20–40 nm, in combination with the in situ and deliberately created hole along the radical direction, enables monochromatic laser‐driven lighting. The size rationale of silica aerogel building blocks, lies in regulating the concentration of surfactant in the mixture with aqueous acetic acid for the condensation reaction. The implementation of three primary color lasers (wavelengths of 450, 532, and 638 nm) with variant powers on the silica aerogels can realize the lighting with multiple colors in the visible spectra. It can be anticipated that, building silica aerogel fibers with good thermal stability, high laser‐damage threshold, and superhydrophobicity can find applications in remote laser‐driven lighting textiles even on rainy days or underwater circumstances, curtain lighting, smart display system, or digital signage.^[^ ^30^, ^31^ ^]^

### 4.5 Fire‐Retardant Properties

Aside from other properties, some aerogel fibers could exhibit superb fire‐retardant properties, which are attributed to both the inherent molecular rigidity and high cohesive energy density. For example, Kevlar aerogel fiber shows outstanding flame‐retardant properties and self‐extinguishing behavior.^[^ ^13^ ^]^ The other example is polyimide aerogel fiber, where the backbone of the polymer chain contains amounts of benzene and imide rings. The self‐extinguishing performance of the above single aerogel fiber and the textiles has been investigated, which can be employed for fire‐retardant clothing.^[^ ^13^, ^21^ ^]^

**Figure 12:**
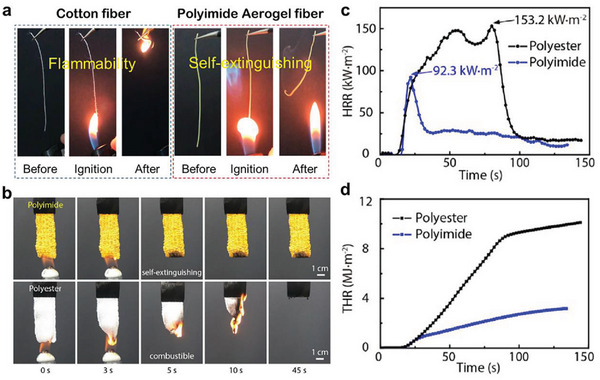
Fire‐retardant properties of aerogel fibers. a) The superb flame resistance of polyimide aerogel fiber compared with cotton fiber. Reproduced with permission.^[^ ^21^ ^]^ Copyright 2021, American Chemical Society. b) The burning test of polyimide and polyester textiles. The polyimide textile keeps intact while the polyester textile is completely combusted. c) Heat release curve of the polyimide and polyester textiles. d) Total heat release curves of the polyimide and polyester textiles. (b–d) Reproduced with permission.^[^ ^29^ ^]^ Copyright 2020, Elsevier B.V.

During the ignition test, the cotton fiber is easily ignited approaching the fire and almost burnt up within 1 s, even quickly moved away from the fire. Conversely, the polyimide aerogel fiber cannot be ignited in a short touch on the fire and shows immediate self‐extinguishing performance after keeping away from the flame (**Figure** **12**a). The limiting oxygen index of polyimide aerogel fiber is determined as 46.2, leading to better flame resistance than other polymers, such as cotton and Kevlar, with a limiting oxygen index of 24 and 28, respectively.^[^ ^21^ ^]^

When both polyimide and polyester textiles were ignited by the flame, polyimide textile could be rapidly self‐extinguished upon the removal of the flame; on the contrary, the polyester textile was completely combusted (Figure 12b). Furthermore, the cone calorimetry test was conducted to quantitatively characterize the fire‐retardancy of both materials. The polyimide aerogel textile has a lower value of heat release rate (92.3 kW·m^−2^) than that of polyester textile (153.2 kW·m^−2^) (Figure 12c). Upon the ignition, the materials with a higher heat release rate tend to be more dangerous as more heat will be fed back to the surface of materials causing accelerated thermal decomposition. As a result, the total heat release of polyester textile (10.1 MJ·m^−2^) outweighs the polyimide textile (3.2 MJ·m^−2^) (Figure 12d). In addition, the commercial cotton textile is characterized, which shows significantly inferior thermal insulation properties than that of polyimide textile.^[^ ^29^ ^]^

Above all, aerogel fibers have the mass and surface distributions that are consistent with fractal behavior over certain length scales. To fully unveil the structure/property relationships, it is necessary to let the experimental study go hand‐in‐hand with the simulation methods. In a computational model, the aerogel fiber structure is known exactly; and therefore, the structure–property relationships can be directly understood. For example, global parameters such as surface areas, fractal dimensions, and pore size distributions can be determined from the aerogel model structure and compared with the experimental data. Microscopic parameters including the number of bridging atom to silicon atom (e.g., SiO_2_ aerogel fiber), the bond lengths, and bond angles are also measured. Last, the mechanical properties including the modulus, the vibrational density of states, and shrinkage upon drying can be correlated with the gel structure. Similarly, other properties can also be derived based on the simulated methodology including quantum mechanical methods, atomistic simulations and “coarse‐grained” models.^[^ ^66^ ^]^

## 5 Nano‐Confining Functionalization Strategy

**Figure 13:**
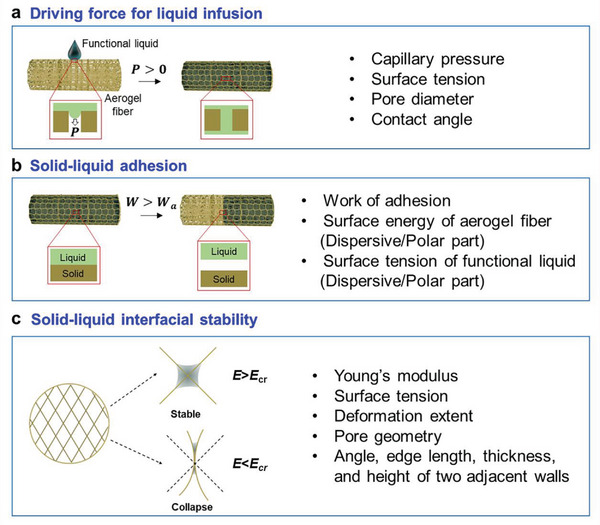
Driving force, solid–liquid adhesion, and solid–liquid interfacial stability of the nano‐confining functionalization strategy for various aerogel fibers. a) Driving force for liquid infusion. b) Solid–liquid adhesion to ensure the long‐term stability. c) Solid–liquid interfacial stability.^[^ ^106^ ^]^

Aside from the inherent properties of aerogel fibers as solid porous materials, it could be an alternative way to enrich the functionalities of aerogel fibers, by utilizing the high porosity and high specific pore volume of the fibers to incorporate with some guest materials in the pore structures. Especially for liquid materials, the liquid itself cannot be used as a freestanding material due to the fluidity, no fixed shape, leakage, and weak mechanical properties.^[^ ^95^ ^]^ However, aerogel fibers can be advantageously used to provide the nano pockets for functional liquids with the capillary force that ensures the stay of infused liquid. Through the rational design of aerogel fiber materials, functional liquid materials, and the chemical compatibility of the solid–liquid interface, a huge pool of aerogel fiber‐confined solid–liquid composite materials would be generated, enabling unprecedented properties and applications. Consequently, despite the variety of functionalization strategies for aerogel fiber, for instance, hydrophobic modification,^[^ ^13^, ^22^, ^26^ ^]^ hygroscopic modification,^[^ ^9^ ^]^ and color modification,^[^ ^13^ ^]^ the nano‐confinement of functional liquids into mesopores of the aerogel fibers becomes more and more attractive due to the properties enabled by the liquid such as phase change,^[^ ^15^ ^]^ sorption,^[^ ^9^, ^96^ ^]^ transparency,^[^ ^97^, ^98^ ^]^ slippery,^[^ ^99^ ^]^ self‐healing,^[^ ^96^, ^98^ ^]^ frictionless,^[^ ^100^ ^]^ antifouling,^[^ ^101^, ^102^ ^]^ anti‐adhesion,^[^ ^103^ ^]^ and anti‐icing,^[^ ^104^, ^105^ ^]^ as well as the interaction between the solid aerogel fiber and the liquid guest.^[^ ^26^, ^106^ ^]^ In this section, we will mainly introduce the driving force for liquid infusing, solid–liquid adhesion, and solid–liquid interfacial stability (**Figure** **13**). Currently, the representative examples of functional liquids that are packaged into the aerogel fibers are phase change materials and water.^[^ ^9^, ^13^, ^15^, ^23^, ^26^, ^29^ ^]^

### 5.1 Driving Force for Liquid Infusion

Nano‐confining functionalization of aerogel fibers by functional liquids is tightly related to the thermodynamics and kinetics of liquid encapsulation in the porous framework. How to obtain the aerogel fiber solid–liquid composites and how fast can they form? To package the liquid in the aerogel fiber structure, we can infuse the functional liquid into the porous solid or immerse the porous solid into a pool of the functional liquid. The driving force for the liquid infusion depends on the capillary pressure (Figure 13a), which can be described as,^[^ ^69^ ^]^

(10) $$P\;=\frac{2\gamma cos\theta }{d}\;$$

where, *P* is the capillary pressure, *γ* is the surface tension of the functional liquid, *d* is the pore diameter of the aerogel fiber, and *θ* is the contact angle between the liquid and solid phases. Immersing the aerogel fiber into a liquid generally liberates heat; the enthalpy of immersion can be written,^[^ ^107^ ^]^

(11) $$\begin{array}{ccc} h_{i} & = & \gamma_{\mathrm{SL}}\;-\gamma_{S}-T\;\left(\frac{\partial \gamma_{\mathrm{SL}}}{\partial T}-\frac{\partial \gamma_{S}}{\partial T}\right)\\ & = & -\;\gamma_{\mathrm{LV}}\cos\theta -\left(\gamma_{S}-\gamma_{\mathrm{SV}}\right)-T\left(\frac{\partial \gamma_{\mathrm{SL}}}{\partial T}-\frac{\partial \gamma_{S}}{\partial T}\right) \end{array}$$

where, *γ*_SL_, *γ*_S_, *γ*_LV_, and *γ*_SV_ denote the solid–liquid interfacial tension, solid surface energy, liquid‐vapor interfacial tension, and solid‐vapor interfacial tension, respectively, *T* is the temperature, and *θ* is contact angle. As in practice, *γ*_S_ − *γ*_SV_ is negligible, and low energy surfaces have a relative constant value of $\frac{\partial \gamma_{\mathrm{SL}}}{\partial T}=\;0.07\;\pm \;0.02\;\mathrm{erg}\;cm^{-2}\;K^{-1}.$ A simple relationship can be obtained as,^[^ ^107^ ^]^

(12) $$h_{i}=\;-0.07T-\gamma_{\mathrm{LV}}cos\theta$$

Polar solids show high enthalpy of immersion in polar liquids and lower values in nonpolar liquids. *h*_i_ is principally a linear function of the dipole moment of a functional liquid.

Regarding the kinetics of liquid infusion, it involves the flow of the functional liquid over the aerogel fiber, and it refers to the speed with which the solid–liquid‐vapor three‐phase line advances or recedes. Typically, the dynamic contact angle can be measured to obtain the speed of the three‐phase line. A dimensionless parameter to characterize the speed of contact line movement is the capillary number *Ca*,^[^ ^107^ ^]^

(13) $$Ca\;=\frac{v\eta }{\gamma_{\mathrm{LV}}}\;$$

where, *v* is the velocity, *η* is the fluid viscosity, and *γ*_LV_ is the liquid–vapor interfacial tension (i.e., surface tension of the functional liquid).

### 5.2 Solid–Liquid Interface Adhesion

Ideally, the functional liquid should firmly adhere to the aerogel fiber, eliminating leakage or vaporization. Adhesion between a solid material and a liquid material is defined as the work needed to separate the solid from the liquid.^[^ ^107^ ^]^ The important quantity work of adhesion could be used to characterize the intimate contact between the liquid phase and solid phase (Figure 13b).^[^ ^107^, ^108^, ^109^, ^110^, ^111^ ^]^ This is largely determined by the surface energy of the solid material and the surface tension of the functional liquid, that is, it is the chemistry compatibility between the solid and liquid interface. According to the method of Owens, Wendt, Rabel, and Kaelble (OWRK), the interfacial energy between the solid and liquid can be calculated by forming the geometric mean:^[^ ^112^ ^]^

(14) $$\sigma_{\mathrm{sl}}=\sigma_{s}\;+\sigma_{l}-2\left(\sqrt{\sigma_{s}^{p}\sigma_{l}^{p}}+\sqrt{\sigma_{s}^{d}\sigma_{l}^{d}}\right)$$

The work of adhesion based on the method of OWRK is,^[^ ^112^ ^]^

(15) $$W_{A}=\;2\left(\sqrt{\sigma_{s}^{p}\sigma_{l}^{p}}+\sqrt{\sigma_{s}^{d}\sigma_{l}^{d}}\right)$$

To obtain work of adhesion, it is necessary to determine the dispersive part and polar part of the solid surface energy (aerogel fiber material), as well as the dispersive part and polar part of the liquid surface tension. For example, a functional liquid with the surface tension of 27.91 mN m^−1^ (dispersive part: 25.98 mN m^−1^ and polar part 1.98 mN m^−1^) can adhere well to a solid copper foil with a surface energy of 23.89 mN m^−1^ (dispersive part: 22.34 mN m^−1^ and polar part 1.55 mN m^−1^), possessing the work of adhesion of 51.64 mN m^−1^.^[^ ^109^ ^]^ To optimize the work of adhesion, two approaches can be considered: on the one hand, it is possible to modify the liquid in respect of its polar versus dispersive ratio; on the other hand, it is likely to increase the polarity of the solid surface by treatment, for example, plasma treatment.

### 5.3 Solid–Liquid Interfacial Stability

If any ingredients can soften or swell the solid frame, the aerogel fiber may encounter shrinkage or collapse. It is important to make sure that the functional liquid preferentially wets the pore walls rather than swells them. Therefore, the aerogel frame should be able to withstand the infusion and wetting of the liquid, that is, it should have robust structural stability and mechanical properties. To simplify the model of an aerogel fiber, we can focus on the two adjacent pore walls forming an angle *α*, with an edge length of *l*, the thickness of *t*, and the height of *h*. Once the functional liquid is applied to the pore structure, the formation of a series of networks of menisci on the interconnected pores generates the localized capillary force field at each node. Work done by capillarity can be determined by the product of the capillary force and the distance moved by the meniscus,^[^ ^106^, ^113^, ^114^, ^115^ ^]^

(16) $$W_{c}=\;\gamma hl\sec\frac{\alpha }{2}$$

where, *γ* is the surface tension of the functional liquid. The total elastic energy of a single pore wall can be determined by the sum of the related stretching and folding,^[^ ^106^ ^]^

(17) $$U_{e}=\frac{1}{2}\;E_{\mathrm{wet}}h\times \left[{\left(sec\frac{\alpha }{2}-1-\delta \right)}^{2}lt+\frac{1}{3}\alpha t^{2}{\left(1+\delta \right)}^{2}\right]$$

where, *E*_wet_ represents Young's modulus of the material in a wetting/swollen state, and *δ* is the swelling ratio (deformation extent) by the functional liquid. In order to avoid the deformation of the pore structure, the capillary work should be smaller than the elastic energy, that is, *W*_c_ < *U*_e_. Such energy relationship derives a critical Young's modulus of the aerogel fiber material, characterizing the capability to resist pore wall deformation,^[^ ^106^ ^]^

(18) $$E_{\mathrm{cr}}=\frac{6\gamma l\sec\frac{\alpha }{2}}{3lt{\left(\sec\frac{\alpha }{2}-1-\delta \right)}^{2}+ab^{2}{\left(1+\delta \right)}^{2}}\;$$

When the modulus of aerogel fiber material is higher than this critical value, the fiber can stably keep its form; otherwise, it may suffer from a structural collapse in contact with the liquid (Figure 13c). Key features in establishing an aerogel fiber‐functional liquid combination that can ensure the pore network stability include the basic properties of aerogel fiber material and the functional liquid. The properties of aerogel fiber materials include the chemical nature, crystallization, crosslinking density, porosity, and topology. The properties of the functional liquid include the solubility, volatility, and Flory–Huggins polymer–solvent interaction parameter.^[^ ^106^, ^116^, ^117^ ^]^

## 6 Emerging Applications

In light of the unprecedented properties mentioned in the previous sections, such as thermal, sorptive, optical, and mechanical properties, aerogel fibers have enabled a myriad of emerging applications in the fields of thermal management, smart wearable fabrics, water harvest, shielding, heat transfer devices, artificial muscles, and information storage.

### 6.1 Thermal Management

**Figure 14:**
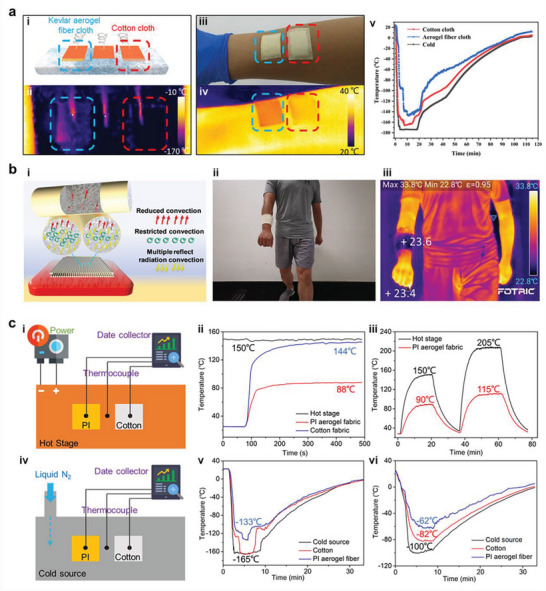
Thermal management. a) Kevlar aerogel textile for thermal insulation. Kevlar aerogel textile and cotton textile were tested under low temperature, on human skin, and the temperature–time curve of different surfaces with the injection of liquid N_2_. Reproduced with permission.^[^ ^13^ ^]^ Copyright 2019, American Chemical Society. b‐i) Polyimide aerogel textile for thermal insulation. Schematic illustration of the insulation mechanism, b‐ii) optical, and b‐iii) infrared images of the fabric on a human arm. Reproduced with permission.^[^ ^24^ ^]^ Copyright 2021, American Chemical Society. c) Thermal insulation performance of polyimide textile on c‐i–iii) hot stage and c‐iv–vi) cold source compared with cotton textile. Reproduced with permission.^[^ ^21^ ^]^ Copyright 2021, American Chemical Society.

Thermal management is by far the most representative and studied application field of aerogel fibers due to the ultralow density and ultrahigh porosity. For example, Kevlar textiles woven from aerogel fibers exhibit superior thermal insulation performance compared with cotton textiles with the same thickness, when both of them are attached to the human skin (**Figure** **14**a‐iii,iv), and serviced on a cold source (−196 °C to 20 °C, Figure 14A,i‐ii) or hot source (20 °C to 350 °C). At the equilibrium state on the cold surface, Kevlar aerogel textile displays a higher temperature than cotton textile. With the injection of liquid N_2_, the surface temperature of the cold source decreases until the equilibrium of −190 °C, whereas the surface equilibrium temperatures of aerogel textile and cotton textile are −148 °C and −168 °C, respectively. The higher temperature difference for aerogel textile (42 °C) indicates better thermal insulation properties. Moreover, Kevlar aerogel textiles can withstand heat treatment, while other commercial ones including cotton textiles or microfiber mats are severely damaged if heated to 300 °C. Moreover, Kevlar aerogel fiber also shows an outstanding flame‐retardant effect and self‐extinguishing behavior. The superb thermal insulation performance, combined with flame‐retardant behavior, provides aerogel fibers with the potential application for thermal insulation under harsh conditions, for example, fire protective clothing.^[^ ^13^ ^]^

Other examples will be drawn from polyimide aerogel fibers, which also exhibit super‐thermal insulation and flame resistance that allow for thermoregulation in extreme environments.^[^ ^21^, ^24^, ^29^ ^]^ The gas molecules cross polyimide fibers in the textile with small pores (15–25 nm) do not subject to collisions and exchange energy with each other; thus greatly restricting thermal convection. The high porosity of polyimide aerogel textiles can effectively reduce thermal conductivity. Besides, the random pores and nanofibrils may lead to multiple reflective effects; so that, the reflectance of infrared light would be largely improved and thermal radiation would be reduced (Figure 14b‐i). Once the polyimide aerogel textile is attached to human skin, the surface temperature of the textile is almost close to that of the background, indicating the potential application as thermal shielding/stealth materials (Figure 14b‐ii‐iii). The textile can be applied over a broad range of temperatures (−190 °C to 320 °C), ranking as the state‐of‐the‐art thermal insulation materials.^[^ ^24^ ^]^

Polyimide aerogel textile can also withstand both elevated and low temperatures, enabling its utilization in extreme circumstances. When placed on the heating source at 150 °C, polyimide aerogel textile takes a longer time to reach the equilibrium state (144 °C) than cotton textile (88 °C), displaying better thermal insulation performance. Meanwhile, it also possesses a higher temperature difference referenced to the heating stage, surpassing the performance of cotton textiles (Figure 14c‐i‐ii). If the heating source is set to a higher temperature (205 °C), polyimide aerogel textile still keeps the insulation function, while a series of commercial polymeric textiles including cotton easily fail in such harsh conditions (Figure 14c‐iii). Furthermore, polyimide aerogel textiles can survive at low temperatures owing to the low‐temperature reliability of the polyimide molecular structure. As low as −165 °C, polyimide aerogel textile can be stabilized at such a temperature, whereas the compared cotton textile can only reach −133 °C. When the cold source is at −100 °C, the equilibrium of polyimide aerogel textile and cotton textile can reach −62 °C and −82 °C, respectively, further demonstrating a superb thermal endurance at low temperatures.^[^ ^21^ ^]^

### 6.2 Smart Wearable Fabrics

**Figure 15:**
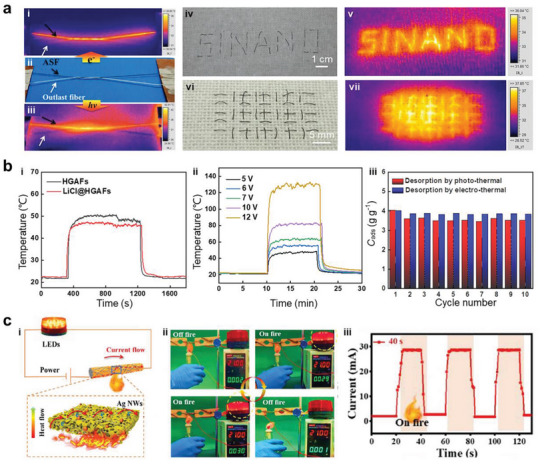
Smart wearable fabrics. a) Graphene aerogel/phase change material fibers show multiple responsiveness under electric field and light irradiation. Reproduced with permission.^[^ ^15^ ^]^ Copyright 2018, Wiley‐VCH. b) Hygroscopic holey graphene aerogel fibers can be regenerated by photo‐thermal and electro‐thermal approaches with fairly stable cycle stability. Reproduced with permission.^[^ ^9^ ^]^ Copyright 2022, Springer Nature. c) Calcium alginate/Fe_3_O_4_ nanoparticles/Ag nanowires self‐powered fire alarm electronic textile. c‐i) The schematic of the mechanism, c‐ii) thermally triggered fire alarm, and c‐iii) cyclic responsive curve of the e‐textile. Reproduced with permission.^[^ ^28^ ^]^ Copyright 2022, American Chemical Society.

Smart aerogel fibers in response to external stimuli, such as thermal, electrical, and light fields, as well as with energy storage/conversion capability, are crucial units for wearable devices. For instance, the graphene aerogel/phase change material (GA/PCM) fibers show multiple responsiveness (e.g., electrical or photonic) with a broad range of programmable phase transition temperatures and enthalpy. When the GA/PCM fiber is twined with outlast fiber under the electric field of 30 V and one sun irradiation, there is more heat generated around the GA/PCM fiber, demonstrating superior electric–thermal and photo–thermal effects. A variety of patterns can be developed with the GA/PCM fibers such as the “SINANO” latter pattern and network weaved fabrics with other materials, which can be illuminated by light irradiation. The GA/PCM bundle and GA/PCM fabrics exhibit faster heating rate and higher temperature compared with single GA/PCM fiber, illustrating quicker heat transfer and less heat loss happens in the GA/PCM bundle and the fabrics. It means that the graphene network plays a central role in electron transfer, phonon transfer, and heat transfer in both inducing and facilitating the phase transition of phase change materials confined in the pore structure (**Figure** **15**a).^[^ ^15^ ^]^ The superhydrophobic coating on the fiber enables self‐cleaning behavior and enhanced mechanical properties. These features together promise aerogel fibers for flexible and wearable devices, especially for clothes in frigid areas.

Besides, the hygroscopic holey graphene aerogel fibers (LiCl@HGAFs) have been developed with photo–thermal and electro–thermal responses. Indeed, the LiCl@HGAFs show high water sorption capacity as well as high water uptake kinetics, which can be regenerated by solar energy and electric energy and both of them are environmentally‐friendly regeneration processes. The HGAFs and LiCl@HGAFs exhibit a fast photothermal effect under one‐sun irradiation, where the temperature rises from 22 °C to 46 °C and 44 °C, respectively (Figure 15b‐i). In this condition, the sorbent experiences the desorption from LiCl aqueous solution to LiCl·H_2_O with a regeneration degree of 83.4%. On the other hand, the fibers can be desorbed by the electric field, through which the temperature can be up to 131 °C under 12 V (Figure 15b‐ii). In this case, the sorbents are almost completely desorbed. It is noteworthy that the fibers can undergo multiple cycles (up to ten) of sorption and desorption without any obvious degradation via the photo–thermal and electro–thermal approaches (Figure 15b‐iii).^[^ ^9^ ^]^ Furthermore, the self‐powered fire alarm electronic textiles (SFA e‐textile) have been reported based on the aerogel fibers comprised of calcium alginate, Fe_3_O_4_ nanoparticles, and Ag nanowires. The fire alarm trigger time decreases with increasing the amount of Ag nanowires, which improves the sensitivity of the warning system. This is attributed to the fact that the nanowires can be connected, creating a continually conductive pathway on the surface of the fiber (Figure 15c‐i). The e‐textile can be repeatedly induced by the fire and rapidly trigger the fire alarm system once it is exposed to a flame (Figure 15c‐ii). The e‐textile is exposed to a fire at cycle intervals of 20 and 40 s, while the electric current is monitored simultaneously. An average current of 28 mA with a small standard deviation (5%) is obtained, ensuring fairly stable and repeatable fire alarming capability of the textile (Figure 15c‐iii). The stable performance is due to the conductive stability of Ag nanowires at elevated temperatures. SFA e‐textile can realize ultrasensitive temperature monitoring with a wide range of sensing and energy harvesting in firefighting clothing. The ultralight wearable temperature‐monitoring SFA e‐textile can be of great help for rescuers to search and rescue trapped firefighters in fire conditions.^[^ ^28^ ^]^

### 6.3 Water Harvest

**Figure 16:**
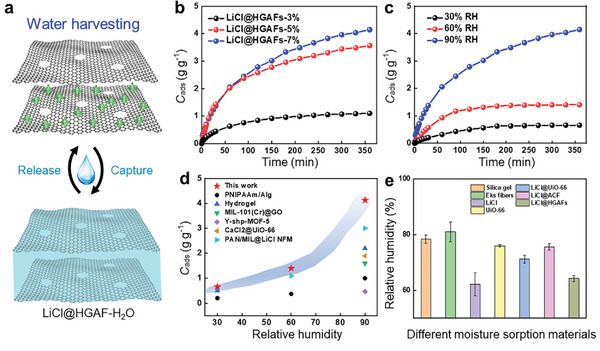
Highly efficient water capture by hygroscopic holey graphene aerogel fibers. a) Schematic illustration of water capture and release. b) Water uptake capacity of LiCl@HGAFs with variant salt concentrations. c) Water uptake capacity of LiCl@HGAFs under different levels of humidity. d) Comparison of water capture capability materials from literature. e) Dehumidification test of LiCl@HGAFs compared with a series of moisture sorption materials in the same mass. a–e) Reproduced with permission.^[^ ^9^ ^]^ Copyright 2022, Springer Nature.

With hygroscopic salt as the guest species to be anchored in the porous network, aerogel fibers can find promising applications for water harvest, such as atmospheric water capture. As demonstrated before, the hygroscopic holey graphene aerogel fibers (LiCl@HGAFs) achieve both the water capture capacity and high uptake kinetics due to the abundant pathways in holey graphene sheets for water transport. The content of hygroscopic salt plays a vital role in the moisture sorption capability, which increases with increasing the loading amount of the salt (**Figure** **16**b), with the highest uptake capacity of 4.14 g g^−1^ (salt content of 7%). In addition, the LiCl@HGAFs can work in a broad range of humidity from 30% RH to 90% RH (Figure 16c), with the water capture of 1.4 and 0.66 g g^−1^ at the moisture humidity of 60% RH and 30% RH, respectively. The sorption capability of the as‐developed aerogel fibers outperforms other moisture sorbents from literature, achieving more than 30% above the best material reported at room temperature and 90% RH (Figure 16d). When dehumidification tests are conducted, the aerogel fiber demonstrates competitive performance, together with a series of moisture sorption materials either in the same volume or in the same mass. For the competitive test with the same mass, LiCl@HGAFs surpass most of the other sorbents but is slightly inferior to LiCl. (Figure 16e). Therefore, LiCl@HGAFs can meet the needs of practical applications because they can be operated at ambient temperatures and regenerated in multiple pathways with low‐energy input.^[^ ^9^ ^]^

### 6.4 Shielding

**Figure 17:**
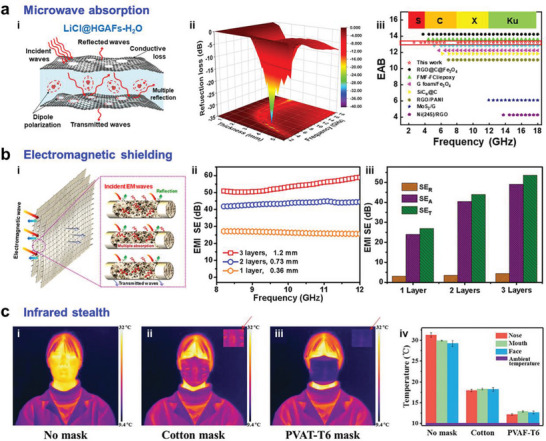
Shielding applications of aerogel fibers. a) Microwave absorption by hygroscopic holey graphene aerogel fibers. Reproduced with permission.^[^ ^9^ ^]^ Copyright 2022, Springer Nature. b) Electromagnetic shielding by polyimide aerogel fibers. Reproduced with permission.^[^ ^24^ ^]^ Copyright 2021, American Chemical Society. c) Infrared stealth is enabled by poly(vinyl alcohol) aerogel fibers. Reproduced with permission.^[^ ^47^ ^]^ Copyright 2021, Wiley‐VCH.

Utilizing the porous nature, electrical conductivity, and interface effect, aerogel fibers could be tailored for microwave adsorption or electromagnetic shielding. For example, with the filling of LiCl particles in the holey graphene sheets, the layered graphene sheets lead to multiple microwave reflections and the conductivity of holey graphene sheets induces conductivity loss; in combination with the interface polarization between the graphene and LiCl, the microwave sorption performance is greatly enhanced. It is worth noting that, after the water capture by hygroscopic LiCl particles, the dielectric loss of the material is further increased and dipole polarization from water molecules is also induced, endowing LiCl@HGAFs with broad microwave absorption behavior (**Figure** **17**a‐i,iii). After the sorption of water, LiCl@HGAFs‐H_2_O can absorb the microwave spanning from 8.31 to 18 GHz with a sample thickness of 2.5 mm. Meanwhile, the minimum reflection loss of the material is −27.9 dB at 17.3 GHz (Figure 17a‐ii). LiCl@HGAFs‐H_2_O exhibits broad microwave sorption capability with the effective absorption bandwidth (EAB) covering all of the Ku, X, and C bands, and partial S bands (Figure 17a‐iii).^[^ ^9^ ^]^

Apart from that, decorating metal nanoparticles on polymer‐based aerogel fibers provides an alternative pathway to develop lightweight conductive fibers for electromagnetic shielding applications. Ag nanoparticles^[^ ^24^ ^]^ or Ni nanoparticles^[^ ^37^ ^]^ have been reported to decorate the surface of polymer aerogel fibers by electroless plating; the resulting composite conductive aerogel fibers, as well as their woven textiles, exhibit superb electromagnetic shielding performance (Figure 17b).^[^ ^24^ ^]^ When the electromagnetic wave injects the surface of the textile, only a shallow part of the incident wave is reflected encountering the Ag nanoparticle layer. The rest of the electromagnetic waves travel through the textile and interact with the electron carriers. Specifically, the high porosity of the fiber can induce multiple internal interactions; and thus, resulting in the dramatic adsorption of electromagnetic waves (Figure 17b‐i). The single‐layer textile has an EMI SE of 26 dB, which is increased to 44 and 54 dB when layering two and three pieces of the textile at 10 GHz (Figure 17b‐ii). For the total EMI shielding effectiveness (SE_T_), microwave absorption (SE_A_) makes more contribution than microwave reflection (SE_R_), demonstrating the absorption‐dominated EMI shielding mechanism (Figure 17b‐iii).^[^ ^24^ ^]^

Besides, aerogel fibers (e.g., poly(vinyl alcohol)) have also been proposed for infrared stealth due to their extraordinary thermal insulation. With such materials, the infrared radiation intensity of a certain target can be efficiently decreased, making the temperature difference undetectable by an infrared camera (Figure 17c).^[^ ^47^ ^]^ Based on the infrared images of a person without a mask, with a cotton mask, and a PVA aerogel textile mask, the color of the PVA aerogel textile mask is almost merged with the background. Therefore, by rationalizing the content and structure, aerogel fibers can be designed for shielding purposes, including microwave adsorption, EMI shielding, and infrared stealth.

### 6.5 Heat Transfer Devices

**Figure 18:**
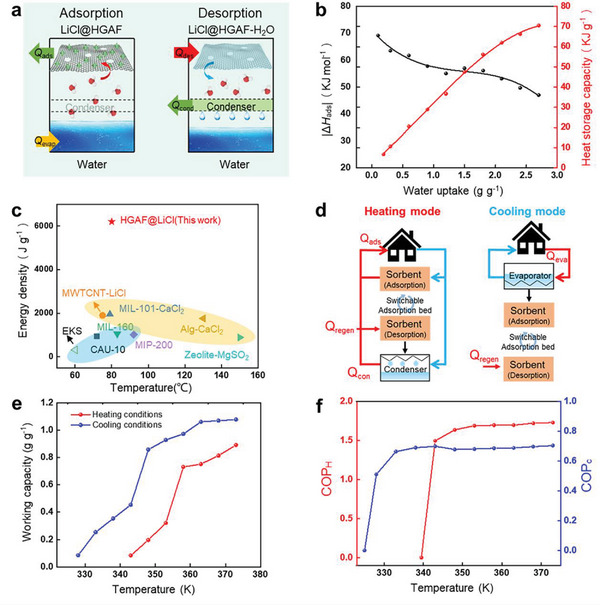
Heat transfer devices. a) Mechanism of LiCl@HGAFs applied in adsorption‐based heat transfer device. b) Isosteric enthalpy of adsorption and heat storage capacity for water. c) Comparison of energy density among different sorbents. d) Heat transfer is related to the heating and cooling modes. e) Working capacity for heating and cooling conditions. f) The coefficient of performance (COP) for heating and cooling at variant driving temperatures. a–f) Reproduced with permission.^[^ ^9^ ^]^ Copyright 2022, Springer Nature.

For the holey graphene aerogel fibers mentioned before, in addition to mass transfer during the water sorption/desorption process, heat transfer coexists in terms of heating and cooling correspondingly. Therefore, aerogel fibers have been well played in the adsorption‐based heat transfer devices by incorporating the efficient “sorbent‐water” working pair. During the adsorption process, the evaporated working fluid of water is captured by aerogel fibers (LiCl@HGAFs) and releases heat (heat of adsorption, *Q*_ads_). Conversely, in the desorption process, the aerogel fibers take the heat (heat of desorption, *Q*_des_) from the environment to release the water, which condenses later in the condenser releasing heat (heat of condensation, *Q*_cond_) (**Figure** **18**a).^[^ ^9^ ^]^ The adsorption‐based heat transfer devices such as adsorption‐driven heat pumps, adsorption‐driven chillers, and thermal batteries, have been considered cutting‐edge renewable energy solutions to satisfy the huge global demands for heating/cooling.

Along with the water sorption, the isosteric enthalpy of sorption (|Δ*H*_ads_|) can be determined based on the Clausius–Clapeyron equation from two adsorption isotherm curves. Moreover, the heat storage capacity can be obtained from the isosteric enthalpy of sorption, water molecular weight, and sorption working capacity. |Δ*H*_ads_| decreases with increasing water uptake, with the value of 63 kJ mol^−1^ at the working capacity of 0.6 g g^−1^. The heat storage capacity increases with uptake capacity (Figure 18b).^[^ ^9^ ^]^ The heat storage capacity is 6.93 kJ g^−1^ (0.19 kW h kg^−1^) at the water uptake of 0.24 g g^−1^, which is 1.68 times higher than the value required by the U.S. Department of Energy (0.07 kW h kg^−1^). Compared with other reported adsorbents, LiCl@HGAFs exhibit a high energy density that is promising for thermal storage applications (Figure 18c).^[^ ^9^ ^]^

Adsorption‐based heat transfer system typically operates by utilizing a full cycle of water adsorption and desorption. In the heating mode, the LiCl@HGAFs sorbents capture water and release the adsorption heat to warm the house. In the cooling mode, the heat is taken away by the evaporation of the working fluid, which is induced by the water uptake of the LiCl@HGAFs sorbents (Figure 18d).^[^ ^9^ ^]^ The working capacity shows an increasing trend with rising temperature, both in the heating and cooling modes. It can be determined as 1.1 g g^−1^ because of the complete desorption at the desorption temperature (Figure 18e).^[^ ^9^ ^]^ The coefficient of performance (COP) is the ratio of output energy divided by the input energy, being a vital indicator of the thermodynamic efficiency of the heating and cooling cycle. A high COP_H_ value means a high energy efficiency in the heating mode. The COP_H_ can reach 1.73 at the evaporation temperature of 288 K, sorption temperature of 313 K, and desorption temperature of 373 K. As a comparison, the highest COP_H_ for salt@silica gel is 1.65 with a desorption temperature of 398–423K, which costs more input energy. On the other hand, the COP_c_ for LiCl@HGAFs is 0.7 with the desorption temperature of 373 K. Besides, the specific cooling power of LiCl@HGAFs can reach 297 W Kg^−1^, surpassing other commercial sorbents such as silica gel (63.4 W Kg^−1^), activated carbon (65.0 W Kg^−1^), and zeolite (25.7 W Kg^−1^).^[^ ^9^ ^]^

### 6.6 Artificial Muscles

**Figure 19:**
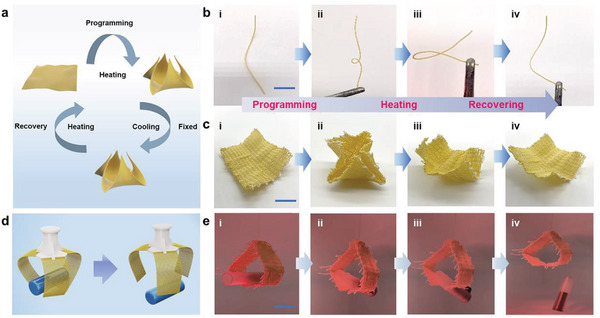
Artificial muscles based on aerogel fibers. a–c) The temperature‐triggered shaper memory effect of phase change wax‐confined aerogel fiber and textile. d,e) A dynamic gripper that can catch and release substance. a–e) Reproduced with permission.^[^ ^26^ ^]^ Copyright 2021, American Chemical Society.

With the incorporation of a phase change material, pristine aerogel fibers can be developed into shape‐memory materials,^[^ ^26^ ^]^ working as artificial muscles.^[^ ^14^, ^118^ ^]^ Taking the Kevlar aerogel fiber as an example, by confining a solid state of paraffin wax into the fiber pores, the bending stiffness of the composite fibers can be modulated above a critical value. As a result, specific artificial muscles with desired shape and shape memory effect can be designed. The target shape is programmed above the phase transition temperature (70 °C) and is fixed at room temperature. For instance, a single paraffin/Kevlar fiber or a textile woven from the fibers with predetermined shapes can stretch out and recover to their original shapes upon heating (**Figure** **19**a–c).^[^ ^26^ ^]^ A dynamic artificial muscle based on composite fibers has been developed, realizing the transportation of an object by gripping it in a rigid state while releasing it in a flexible state. The switch of these two states relies on the phase transition of the phase change materials in the fabrics assembled from the aerogel fibers. The object is captured by two pieces of fabrics at a low temperature below the phase transition point and released upon heating (Figure 19d,e).^[^ ^26^ ^]^ It is worth mentioning that the response time of the artificial muscle could be tailored by controlling the heating power, manipulating the phase change material content, and the assembly of the fibers, arising from the programmable bending stiffness of aerogel fibers.

### 6.7 Information Storage

**Figure 20:**
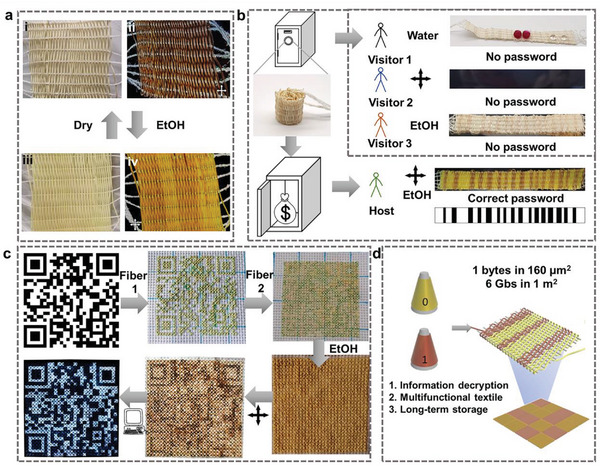
Information storage and decryption based on aerogel fibers. a) Transfer between gel and aerogel by ethanol absorption and ambient pressure drying. b) Barcode information encryption and on‐demand decryption. c) 2D code encryption and on‐demand decryption. d) Advantages of the information storage textile. Reproduced with permission.^[^ ^35^ ^]^ Copyright 2022, American Chemical Society.

Aerogel fibers can hide and reveal information through the modulation of the interaction between a solvent and the aerogel fiber on demand. If properly interweaved, it will enable the production of a digital textile with spatially varying functions. For instance, the Kevlar aerogel fiber with different drawing ratios has been taken as the building blocks for a digital textile. A predesigned pattern is interweaved by these two segments, which is not shown under normal light and polarized light. However, after the impregnation in ethanol, the pattern becomes vividly shown under the polarized light, while it still does not appear under normal light (**Figure** **20**a). This capability of the responsive aerogel fibers demonstrates promising applications in information storage with on‐demand decryption. Interestingly, the coded aerogel fibers can switch the states between aerogel and gel with ethanol adsorption/desorption multiple times (up to ten cycles), possessing good reliability. The specific surface area of the tested fiber is 177 m^2^ g^−1^ after ten cycles of ethanol absorbing and ambient pressure drying (gel‐aerogel switch), slightly lower than that of the initial fiber (204 m^2^ g^−1^). Moreover, only the outer layer of the fiber slightly shrinks but the inferior fiber remains a good porous network after the cyclic operation.

Importantly, more sophisticated patterns such as barcodes and 2‐dimensional (2D) codes could be rationalized by playing with the two different aerogel fibers, making the information readable and processable. The encrypted barcode does not show under normal light or polarized light. After the infusion of ethanol, the information is still protected under normal light. Only in the gel state and with the application of polarized light can the barcode information be identified, and it is even readable and operatable by a computer (Figure 20b). Analogously, a more complicated 2D code with a hidden password is realized by embroidering with two types of aerogel fibers. It is worth mentioning that the encoded information can only be decrypted via ethanol immersion and polarized light observation. After being scanned into a computer, an accurate 2D code would appear for further communication or image processing (Figure 20c). Specifically, the information storage application enabled by aerogel fibers with different building block orientation degrees can represent 0 and 1, constituting a byte via using eight aerogel fibers. Assuming the fiber with a diameter of 10 µm and a byte width of 2 µm is adopted in a digital textile, then, 625 000 bytes can be stored in each square centimeter, that is, 6.0 Gb m^−2^ (Figure 20d). Notably, in terms of information storage when compared with fluorescent, luminescent, or photochromic materials, the aerogel fibers show outstanding advantages such as the ability of binary format information encryption and high security during communication due to the high/low‐temperature stability and two‐step decryption.^[^ ^35^ ^]^

## 7 Challenges and Opportunities

Though aerogel fibers have seen extensive study and fast development, challenges persist regarding how to utilize enormous types of nanoscale building blocks to fulfill the dynamic sol–gel transition, how to realize the industrial‐sale implementation from current lab‐scale operation, how to characterize the thermal and optical properties of the single aerogel fiber, how to manufacture single fiber‐based devices, and to how to promote the intelligence of aerogel fibers with multi‐functionality. With a surge of technological and methodological innovation to overcome the above roadblocks, new opportunities and applications will appear.

### 7.1 Challenges

For the chemical synthesis of aerogel fibers, it remains unresolved to establish a universal strategy for the dynamic sol–gel transition of all kinds of nanoscale building blocks. Hence, chances open for the utilization of machine learning and artificial intelligence, to set up the Materials Genome Initiative of aerogel fibers and reveal some implications for the newcomers. For the industrialization of aerogel fibers, the challenges exist for continuous fabrication, and ambient pressure drying should be of great help depending on the controllable material system. Another challenge is the characterization technique for the thermal/optical properties of a single aerogel fiber. For thermal characterization, it is necessary to seek help from microfabrication that can embed the single fiber with the testing electrode during the fiber assembly. For optical characterization, fluidic assembly within a transparent microfluidic chip that can be easily combined with a spectrometer,^[^ ^119^ ^]^ may play a key role so that the optical properties of the fiber could be captured all the way from the gel fiber to the aerogel one. Similarly, single fiber‐based devices face great challenges due to still weak mechanical properties of most aerogel fibers and integration of the fiber devices.^[^ ^80^ ^]^ Therefore, it is quite urgent to greatly explore new materials for the aerogel fibers used in deformable or portable devices. Last, making the aerogel fibers smarter and smarter is a forever theme. This should be relied on the digging out of the fiber with multiple functionalization in response to several external stimuli or being applied under extreme conditions.

### 7.2 Opportunities

**Figure 21:**
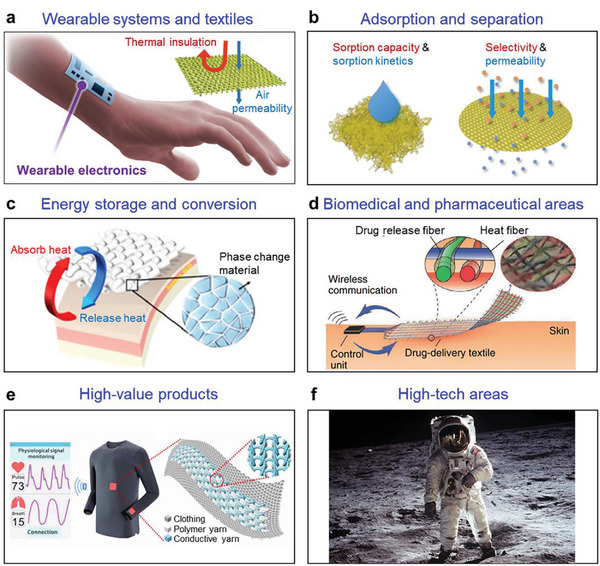
Future opportunities for aerogel fibers. Aerogel fibers can be further deployed in a) wearable systems and textilesReproduced with permission.^[^ ^120^ ^]^ Copyright 2022, Springer Nature. b) Adsorption and separation. c) Energy storage and conversion. Reproduced with permission.^[^ ^121^ ^]^ Copyright 2020, American Chemical Society. d) Biomedical and pharmaceutical areas. Reproduced with permission.^[^ ^122^ ^]^ Copyright 2017, Wiley‐VCH. e) High‐value products. Reproduced with permission.^[^ ^123^ ^]^ Copyright 2019, American Association for the Advancement of Science. f) High‐tech areas. The photo of man in astronaut suit: Reproduced with permission from www.pexels.com, which is free to use.

Opportunities always coexist with the abovementioned challenges. We have witnessed the rapid development of aerogel fibers, and this rising field is going to expand its influence with the continuing efforts of enormous researchers (**Figure** **21**). First, aerogel fibers will continuously play key roles in wearable systems and textiles. Building smart fibers into wearable devices, together with the inherent superb thermal insulation, aerogel fibers will one day flourish in the field of personal thermal management and healthcare management. Importantly, with the porous structure over different length scales, the aerogel textile is a good choice for achieving both excellent thermal insulation and comfortable air permeability as breathable materials. Second, with their porous nature, aerogel fibers may find their tremendous usage in adsorption and separation. Due to the mesopores and micropores in the aerogel fiber, in combination with the macropores between different fibers, it is possible to meet the tradeoff between the fast sorption kinetics and the high sorption capacity for a target species (e.g., ions, molecules, gases, pollutants, etc.). It will also be prone to balance the selectivity and permeability in terms of aerogel fiber‐based membrane separation. Third, a variety of promising biomedical and pharmaceutical applications can be anticipated in the future. Owing to the open pore structure, high specific surface area, and tailored biocompatibility,^[^ ^56^, ^122^ ^]^ aerogel fibers either in the form of fabrics or membranes, are potential candidates for hemostasis or drug delivery systems. Fourth, with the confinement of phase change materials, aerogel fibers can be widely applied for energy storage and conversion. For example, aerogel fiber confined phase change fluid can be used for thermal energy storage, solar energy storage, and heat sink. Fifth, taking the aerogel fiber as the starting material or integrating it with other materials, is likely to offer numerous options for a broad range of functional or high‐value products, such as conductive aerogel fibers, phase change aerogel fibers, biocompatible aerogel fibers, aerogel textile battery, aerogel textile solar cell, porosity‐based heterojunctions, and so on. Last, aerogel fibers shall be underlined for thermal insulation in high‐tech areas, for instance, aeronautical and aerospace domains,^[^ ^10^, ^124^ ^]^ ocean domains, shielding, and beyond.

In addition, these promising opportunities are somehow related to the existing challenges. Addressing the current key challenges would be able to push forward the potential applications. For example, for all the aspects of future opportunities, it is critical to understand the structure–property relationships of aerogel fibers including those of both a single fiber and fiber aggregates. To establish the fundamental structure–property relationships starting from a single fiber, requires unveiling the dynamic sol–gel transition mechanism of nanoscale building blocks. Then, we need to characterize the properties (thermal, optical, sorptive, electric, etc.) of a single aerogel fiber, and further correlate the assembly manner of the fiber with the functionality. This paradigm works for aerogel fibers that can be used in wearable systems and textiles, sorption and separation, energy storage and conversion, high‐tech areas, and so on. Meanwhile, in order to understand the basic function of a single fiber, it is necessary to build the single fiber‐based devices via the microfluidic technology.^[^ ^64^ ^]^ Consequently, single fiber‐based devices can find the applications in biomedical and pharmaceutical areas, such as fiber‐based probes for deep brain activities.^[^ ^125^ ^]^ Furthermore, developing the multi‐functionality capability of aerogel fibers may greatly spur the achievements in high‐value products as well as biomedical and pharmaceutical areas, for instance, the aerogel fibers integrated with either one or all of the following effects: electro–thermal, photo–thermal, and magneto–thermal effects. Ultimately, for all future research directions, the scale‐up fabrication of aerogel fibers is a particular concern because in most applications, huge amounts of fiber aggregates are greatly needed, especially for thermal insulation in textiles and high‐tech areas. The scale‐up implementation is tightly related to both the spinning technologies and drying technologies, which may induce the fully continuous production manner with “nanoscale building blocks in‐aerogel fibers out”.

Last but not the least, the field of aerogel fibers is just at a blossom stage in its evolution. It will steadily leap forward with the development of materials and fabrication technologies. Undoubtedly, the collaboration of interdisciplinary pathways from many fields such as materials science, chemistry, fluid dynamics, textile engineering, and artificial intelligence, will be a logical future direction in aerogel fibers.

## Conflict of Interest

The authors declare no conflict of interest.
